# Viral Prevalence and Genomic Xenology in the Coevolution of HzNV-2 (Nudiviridae) with Host *Helicoverpa zea* (Lepidoptera: Noctuidae)

**DOI:** 10.3390/insects14100797

**Published:** 2023-09-30

**Authors:** Luke R. Tembrock, Frida A. Zink, Todd M. Gilligan

**Affiliations:** 1 Department of Agricultural Biology, Colorado State University, Fort Collins, CO 80523, USA; 2 USDA-APHIS-PPQ-Science & Technology, Identification Technology Program, Fort Collins, CO 80526, USA

**Keywords:** evolution of insect genitalia, speciation, *Helicoverpa armigera*, *Helicoverpa zea*, dsDNA viruses, nudivirus, transposable elements, direct repeats

## Abstract

**Simple Summary:**

Most insect species, like most other animal species, host a number of different viruses, and because the number of insect species are so great, the diversity of viruses in insects is expected to be larger than in any other animal lineage. Despite this expectation, few studies are conducted to characterize such viruses and fewer still are carried out to describe the evolutionary dynamics between host and virus. Here we studied a virus that infects an agriculturally important moth species corn earworm (*Helicoverpa zea*) by comparing host and viral genomes. From this comparison it was found that the virus has incorporated several host genes among which the gene cytosolic serine hydroxymethyltransferase is not known from any other virus and thus may provide a unique study system for better understanding the function of this gene. Additionally, undescribed genes were found in both the virus and host that possess sequences originating from bacteria. Lastly the prevalence of the virus was tested across a broad geographic range and found to be present in nearly all host populations tested. Given these findings, more studies should be initiated to characterize insect viruses for novel molecular interactions with possible uses in gene editing and insect biological control.

**Abstract:**

Insect viruses have been described from numerous lineages, yet patterns of genetic exchange and viral prevalence, which are essential to understanding host–virus coevolution, are rarely studied. In *Helicoverpa zea*, the virus HzNV-2 can cause deformity of male and female genitalia, resulting in sterility. Using ddPCR, we found that male *H. zea* with malformed genitalia (agonadal) contained high levels of HzNV-2 DNA, confirming previous work. HzNV-2 was found to be prevalent throughout the United States, at more than twice the rate of the baculovirus HaSNPV, and that it contained several host-acquired DNA sequences. HzNV-2 possesses four recently endogenized lepidopteran genes and several more distantly related genes, including one gene with a bacteria-like sequence found in both host and virus. Among the recently acquired genes is cytosolic serine hydroxymethyltransferase (cSHMT). In nearly all tested *H. zea*, cSHMT contained a 200 bp transposable element (TE) that was not found in cSHMT of the sister species *H. armigera*. No other virus has been found with host cSHMT, and the study of this shared copy, including possible interactions, may yield new insights into the function of this gene with possible applications to insect biological control, and gene editing.

## 1. Introduction

Viruses are ubiquitous biological entities associated with nearly every form of cellular life. Viral genomes are diverse in structure and can consist of single or double-stranded RNA or single or double-stranded DNA (dsDNA), with sizes varying from 2Kb to 2Mb [1,2]. In prokaryotes DNA viruses are the most common, while in eukaryotes RNA viruses are the most common [3,4]. However, diverse dsDNA viruses are also known from eukaryotes, with arthropods hosting a broad spectrum of these viruses [5]. Long-term persistence and high levels of prevalence have been noted among numerous dsDNA viruses in animals, such as lifelong infections from HSV (herpes simplex virus) and papillomaviruses in mammals [6,7]. The highest degree of persistence among dsDNA viruses is known from braconid parasitoid wasps, where entire viral genomes have been integrated into some wasp genomes and remain functionally active in blocking insect host immune responses [8,9]. Relatives of these fully endogenized viruses (Polydnaviridae), the Nudiviridae, have been described from hosts in the arthropod orders Coleoptera, Decapoda, Diptera, Lepidoptera, and Orthoptera [10]. These nudiviruses are similar to dsDNA viruses like HSV regarding persistence, transmission, and prevalence [11]. While studies of Nudiviridae prevalence are few [12], quantification of prevalence is essential to understanding host–pathogen interactions and coevolution therein.

Numerous viruses are known to infect lepidopteran hosts including at least three in the moth *Helicoverpa zea* [13,14]. The “*Helicoverpa zea* Reproductive Virus,” hereafter referred to with the taxonomically correct HzNV-2 (*Helicoverpa zea* Nudivirus strain 2) naming convention (*sensu* [15,16]), is noted as causing gonadal atrophy among some infected individuals. Previous studies [13,17,18] have demonstrated that the deformed genitalia found in sterile forms of *H. zea* are a result of HzNV-2 infection, specifically, females were noted as having a greatly deformed bursa copulatrix [19]. Transmission of HzNV-2 can occur both horizontally, through individuals mating, and vertically, via the female laying infected eggs with some of the offspring being asymptomatic carriers and others symptomatically sterile [18,20]. Lupiani et al. [12] found that, in some populations, a majority of the wild *H. zea* females tested were positive for HzNV-2, but in most cases the infected individuals were fertile, asymptomatic carriers [20]. Furthermore, the occurrence of HzNV-2 varies in different geographic locations of North America, where prevalence ranges from 0 to 69% [12] due to the multiple modes of transmission and inability to cause mortality or immediate sterility among infected individuals [20]. However, the prevalence of HzNV-2 in *H. zea* has not been examined since the work by Lupiani et al., in 1999, and thus it is unknown if factors such as recent introgression with the sister species *H. armigera* in South America [21] and modern farming practices such as widespread use of Bt toxins have altered the prevalence of HzNV-2.

Historical inferences of HzNV-2 prevalence can be drawn from *Heliothis stombleri* Okumura and Bauer, 1969 which was described as a species separate from *Heliothis* (now *Helicoverpa*) *zea* Boddie, 1850. Male *H. stombleri* were distinguished from *H. zea* based on morphological differences in the valvae and absence of cornuti in the vesica of the genitalia. In females, sclerotization of the ostium and significant deformation of the bursa copulatrix and appendix bursae were used to separate the two species. These descriptions of genitalic malformation match those for symptomatic cases of HzNV-2. A year after the description of *H. stombleri*, Hardwick [22] reared a series of *H. zea* from a single female that consisted of a high percentage of sterile moths matching the description of *H. stombleri*. Because both typical and sterile moths were reared from the same female, Hardwick determined that *H. stombleri* was nothing more than an aberrant sterile form of *H. zea*. The two names were formally synonymized by Pogue [23]. Phylogenetic work by Balbi et al. [24] using DNA sequence data from CO1 and EF-1α further confirmed that *H. stombleri* is synonymous with *H. zea*. To this day, individuals matching the *H. stombleri* description are routinely collected (e.g., [24,25], but the mechanism by which these sterile forms of *H. zea* persist in the population has not been thoroughly linked to HzNV-2 symptoms in the taxonomic literature.

Symbiotic microbial interactions are known to mediate numerous host functions as well as the process of speciation [26,27,28]. While viral infections have not historically been viewed as microbial symbioses (e.g., [29], it is becoming increasingly apparent that viruses play an important, even at times beneficial, role in host–genome evolution through horizontal gene or genome transfer (HGT), acquisition of novel function and/or form [30,31,32,33], and an increase in nucleotide diversity via selection on immune system related genes [34,35,36]. Such viral alterations to host genomes are also observed to be important drivers in certain modes of speciation (e.g., [28,37,38]). In a similar manner, acquisition of host DNA by viruses is thought to be important in the evolution of viral genomes and often associated with novel adaptations to the host environment [39,40,41]. Viral mutation rates are higher than eukaryotic mutation rates [42,43], resulting in an increased substitution rate within acquired genes in the viral genome than within those same genes in the host genome. Because of this, determining the timing of gene integration into a viral genome using phylogenetic methods devised for eukaryotes can be difficult. The correct resolution of a rapidly evolving xenologous viral gene with the slowly evolving genes in the host lineage can produce artifactual results caused by long branch attraction [44]. However, recently acquired genes or those undergoing strong selection for a similar function in both host and virus can be resolved in a position close to the host from which they were acquired.

Here we screened individuals of *H. zea* with aberrant genitalia (consistent with *H. stombleri*) using a highly sensitive droplet digital PCR (ddPCR) assay designed to detect and quantify HzNV-2 viral DNA. Additionally, wild caught *H. zea* individuals with normal genitalia were assayed to quantify viral DNA in asymptomatic males from the same collection locations. Lastly, bulk samples from pheromone traps were assayed in order to provide estimates of viral abundance from a large number of individuals throughout the United States. To study the coevolution of host and virus, we identified highly similar DNA sequences shared between them by comparing HzNV-2 to *H. zea* and the closely related taxa *H. armigera* Hübner, 1809 and *Chloridea virescens* Fabricius, 1777, to establish what genes or DNA fragments may have been recently transferred between the host and HzNV-2. We then annotated the relevant sequences to provide insights into the origins and putative functions of these genes in HzNV-2 (e.g., [45,46]). Phylogenetic analyses were employed to infer the timing of gene transfer. Prevalence of HzNV-2 in *H. zea* populations and the rate, type, and timing of DNA transfers can provide insights into the coevolution of nudiviruses and insect hosts.

## 2. Materials and Methods

### 2.1. Helicoverpa Collection and Identification

Adult male *Helicoverpa* were obtained from pheromone trap surveys for *H. armigera* in Colorado and Puerto Rico, USA conducted from 2014–2015 and used for individual testing of agonadal individuals. The genitalia from these specimens, used for individual assays, were dissected following procedures in Brambila [47] and Pogue [23]. In summary, the abdomen was removed from the specimen, soaked in 10% KOH for approximately 60 min, and descaled using small brushes. The genitalia were removed from the abdomen using forceps and the phallus was separated from the valvae. Cornuti counts were used to verify specimens were *H. zea* (≥15 “sets” of cornuti), *H. armigera* (≤9 “sets” of cornuti), or sterile (no cornuti). In some cases, the vesica was everted to examine the number of basal lobes (3 in *H. zea*, 2 in *H. armigera*). Individual (dissected) and bulk (undissected) moths from pheromone traps were obtained from Colorado, Florida, Georgia, Illinois, Indiana, Louisiana, Maine, Mississippi, Missouri, New Mexico, Oregon, Tennessee, Texas, and Puerto Rico, and used for ddPCR testing. Hybrids were attained from the U.S. Department of Agriculture, Forest Pest Methods Laboratory in Buzzards Bay, Massachusetts through controlled crosses between male *H. zea* and female *H. armigera*, while *H. armigera* samples were collected by collaborators in Spain, Australia, and South Africa (Supplementary Tables S1 and S2). The species identity of all dissected agonadal individuals and undissected individuals was confirmed using the real-time PCR assay described in Gilligan et al. [48] or ddPCR for all bulk samples as described in Zink et al. [49]. All specimens were preserved in ethanol and stored at −80 °C.

### 2.2. DNA Extraction

DNA was extracted from male individuals with aberrant or atypical genitalia matching the description for *H. stombleri*. Positively identified specimens of *H. armigera*, *H. zea,* and lab-reared hybrids between these two species were used as controls. Genomic DNA was extracted from the terminal segment of adult abdomens using a Qiagen DNeasy Blood and Tissue Kit (Qiagen, Valencia, CA, USA) following the protocol described in Tembrock et al. [50]. DNA concentration and purity were measured on a NanoDrop 2000 Ver. 1.6 spectrophotometer (Thermo Scientific/NanoDrop, Wilmington, DE, USA). Bulk extractions were conducted following the procedures described in Gloor et al. [51] and modified in Perera et al. [52] and Zink et al. [49,53].

### 2.3. Droplet Digital PCR for Detection of HzNV-2 DNA

After DNA extraction, genomic DNA was digested using HindIII (New England Biolabs, Ipswich, MA, USA) following manufacturer’s instructions, or fragmented using QIAshredder columns (Qiagen) to ensure DNA fragments were sufficiently small for efficient packaging into droplets. The ddPCR assay was then carried out using primers P4-I, P4-II, P13-I, and P13-II (Table 1) that were designed to amplify two separate loci in the HzNV-2 genome [12]. Due to a single nucleotide mismatch in P4-II, all data presented were generated using the P13 primer set.

The ddPCR analysis was performed on a QX200 Droplet Digital PCR system (Bio-Rad Laboratories Inc., Hercules, CA, USA) using EvaGreen intercalating DNA dye to detect positive droplets following the protocol described in Zink et al. [49]. The assay was optimized for use with the primers from Lupiani et al. [12] regarding primer concentration, annealing temperature, and PCR protocol. For individual specimens, 0.2 ng of total DNA (includes virus and host DNA) was used for each reaction. For bulk samples, DNA was not quantified and 2 µL of extract was used per reaction. The final primer concentration for each reaction was 175 nM for each forward and reverse primer. The PCR program used was as follows: (1) 5 min at 95 °C, (2) 1 min at 95 °C, (3) 1 min at 53.5 °C, (4) 1 min at 72 °C, (5) 34 repetitions of steps 2 through 4, (6) 5 min at 4 °C, (7) 5 min at 95 °C, and (8) an infinite hold at 4 °C. Lid temperature was maintained at 105 °C through all cycles with a ramp rate of 2 °C/s between each step. After reading, droplet data were analyzed using ‘definetherain’ [54] to establish the threshold cutoff above which droplets are considered positive.

### 2.4. Genomic Sequence Comparisons

Several search strategies were employed to compare viral and non-viral DNA. First, host and viral genomes were compared using the complete genome of HzNV-2 (GenBank accession JN418988.1) as a query to BLASTn [55] search against whole-genome contigs (employing the whole genome shotgun database for the three complete genome searches) of *H. zea*, *H. armigera*, and *Chloridea virescens* (taxid: 7113, 29058, and 7102, respectively) separately. The following BLASTn settings were employed: program = blastn; wordsize = 11; expect value 0.05; hitlist size = 200; match/mismatch scores = 2,−3; gapcosts = 5,2; low complexity filter = yes; filter string = L,m; genetic code = 1. All searches were performed using the NCBI webserver unless otherwise specified. Hits from each lepidopteran species were grouped based on HzNV-2 genomic location to avoid calling hits as unique that corresponded to the same regions of the HzNV-2 genome. Second, each of the best hits (cutoff e-value ≤ 0.01; Pertsemlidis and Fondon 2001 [56]) from the three lepidopteran genomes were then used as queries to BLASTn search (using above settings) the entire nt collection to crosscheck that hits of similar function were found across species. Third, complete ORF sequences (using [16] ORF boundaries) or intergenic DNA from HzNV-2 that were shared with at least one of the three lepidopteran species (e-value ≤ 0.01) were annotated by BLASTn searches to the nt database. Additionally, PSI-BLASTp [57] searches (for ORFs only) to the non-redundant protein database were employed with the following settings: program = blastp, word size = 3; expect value = 0.05; hitlist size = 500; gapcosts = 11,1; matrix = BLOSUM62; filter string = F; genetic code = 1; window size = 40; threshold = 11; composition-based statistics = 2, and to the clustered nr database using default settings. Lastly, BLASTn searches (using the above parameters for nt searches) were conducted using custom genomic databases from HiRise scaffolded genomes of *H. armigera*, *H. zea*, and hybrid *H. zea* x *H. armigera* (described below). All search results and subsequent annotations were checked against annotations from Burand et al. [16].

### 2.5. Phylogenetic Analyses

Two complementary phylogenetic approaches were utilized, wherein one approach used a consistently taxon-constrained approach and the other employed a similarity-only criteria in making sequence comparisons. In the first approach, the best matches from the BLAST searches were further scrutinized using several phylogenetic methods to infer the origin of sequences in the HzNV-2 genome. Four HzNV-2 ORFs (Hz2V047, Hz2V066, Hz2V023, and Hz2V035) were aligned with genes (cds only, introns omitted) of the same predicted function from the lepidopteran species *Bombyx mandarina*, *Bombyx mori*, *H. armigera*, *Spodoptera litura*, and *Trichoplusia ni*; the dipteran species *Aedes albopictus*, *Anopheles gambiae*, and *Culex quinquefasciatus*; the hymenopteran species *Megachile rotundata*, *Neodiprion lecontei*, and *Orussus abietinus*; and the coleopteran species *Dendroctonus ponderosae* as an outgroup taxon. These species were chosen because they have high quality genomic resources including predicted gene boundaries, functional annotations, and/or represent species from lineages related to *H. zea*. Where needed, follow-up sequencing was conducted to confirm comparisons. Follow-up sequencing included two HiRise assembled draft genomes (*H. armigera*, and an *H. zea* x *H. armigera* hybrid; GenBank accessions to be added after acceptance), an *H. zea* reference genome ([58]; GCA_022581195.1), as well as PCR generated Sanger sequencing described below. From the predicted gene sequences downloaded from GenBank, ORFs were extracted using ORF finder [59] and then aligned using MUSCLE v 3.8.425 [60]. From the alignment, a NJ (neighbor-joining) tree was resolved with Geneious 9.1.8 using a Tamura-Nei genetic distance model, and branch support assessed with 1,000 jackknife replicates. The NJ tree was used as a guide to perform AICc and BIC tests using MODELTEST v 3.7 [61] to determine the best substitution model and rate variation for the Bayesian analyses (Supplementary Table S3). A phylogenetic analysis using MrBayes v 3.2.6 [62] was run with the following parameters: substitution model and rate variation = GTR+I+G; gamma categories = 4; heated chains = 4; chain length = 1,100,000; subsampling frequency = 200; burn-in = 100,000; and an unconstrained branch length prior. Where AICc and BIC model test results differed, an additional run using the HKY85 substitution model was completed to assess differences in branch support and/or tree topology when using different substitution models. Lastly, phylogenetic analyses using the parsimony method were conducted in PAUP v 4.0a168 [63] in two separate runs using a heuristic tree search in one and a branch-and-bound tree search in the other. For both runs, 1000 bootstrap pseudo-replicates starting with a random seed were used to assess branch support.

In the second phylogenetic approach not constrained by taxon, each HzNV-2 ORF was searched in BLAST using the above settings and the top 100 sequences were downloaded and aligned with MAFFT v 7.450 [64,65]. The alignment was edited to remove short sequences and non-isoform duplicates and realigned with MAFFT. The alignment was used to generate a splits network in SplitsTree v 6.0 [66] using the default Hamming distance calculation with splits assessed using 1000 bootstrap replicates. The ORF Hz2V091 was also assessed in similar manner as above but because it is thought to have a diverse microbial origin the same taxon constrained approach was not applied. Instead, ORF Hz2V091 was searched against the BLAST nr/nt database, and the top 250 sequences were downloaded and aligned with MAFFT from which an unrooted NJ tree was generated. The most distant sequence on the NJ tree was used as the outgroup in a Bayesian analysis using MrBayes. All settings for these analyses were the same as outlined above.

### 2.6. PCR Amplification and Sequencing of cSHMT Segments from H. armigera, H. zea, and Hybrids

Because cSHMT has not been described as a host-acquired gene in other dsDNA viruses, we chose to characterize the host copies in greater detail with follow-up sequencing. Primers were designed using Primer 3 v 2.3.7 [67] with default settings and the SantaLucia [68] method for calculating Tm. Primers were designed in homologous regions of the alignment that would amplify both species and span regions where inserts of interest were inferred. Individuals of *H. armigera*, *H. zea*, and laboratory-reared hybrids between the two species were employed to generate additional sequence data to confirm cSHMT alignments from GenBank-attained sequences. DNA extraction and quantification were carried out as stated above. The primer set eventually employed after testing was Hz_SHMT_1738F and Hza_SHMT_24R (Table 1). PCR reactions were run in 50 µL volumes and consisted of 5 µL 10X Ex Taq buffer (Takara Bio, Shiga, Japan), 4.0 µL dNTPs, 200 nM of each forward and reverse primer, 0.25 µL Taq (TaKaRa Ex Taq HS polymerase), and 36.75 µL water with 1 µL of template DNA. Thermocycling for PCR amplification was: (1) 5 min at 95 °C, (2) 30 s at 95 °C, (3) 1 min at 53 °C, (4) 1 min 30 s at 72 °C, (5) 34 repetitions of steps 2 through 4, (6) 72 °C for 7 min, and (7) 11 °C infinite hold with a lid temperature of 105 °C maintained through all cycles. Amplicons were run on 1% agarose gels to check for multiple bands. In lanes with multiple bands consistent with those expected from the alignments, the bands were excised from the gel using gel cutting tips and purified using the QIAquick Gel Extraction kit (Qiagen, Valencia, CA, USA). Purified PCR amplicons were sent to the University of Chicago Cancer Research Center DNA Sequencing Facility for Sanger sequencing using an Applied Biosystems 3730XL DNA sequencer (Applied Biosystems, Foster City, CA, USA). Sequence electropherograms were edited by removal of terminal low-quality calls, assembled into contigs (forward and reverse) using Geneious, and aligned using MUSCLE for comparison to previous alignments from GenBank sequences. The Sanger sequencing-confirmed insert was BLAST searched against the reference *H. zea* genome (GCF_022581195.2) to find other locations in the genome where similar insertion events have occurred. Draft genomes of *H. armigera* and *H. zea* x *H. armigera* were also employed in the detection of insert activity and similar comparisons (under NCBI BioProject ID PRJNA1020878). These HiRise genomes were generated by Dovetail (Dovetail Genomics, Scotts Valley, CA, USA) from CHiCAGO and HiC proximity ligation libraries and sequenced on an Illumina HiSeq X (Illumina Inc., San Diego, CA, USA), after which the HiRise pipeline was employed for scaffolding [69,70,71].

## 3. Results

### 3.1. Specimen Identification, Confirmation of HzNV-2 in Agonadal Specimens, and Viral Prevalence Determined from Individual and Bulk Samples

**Figure 1:**
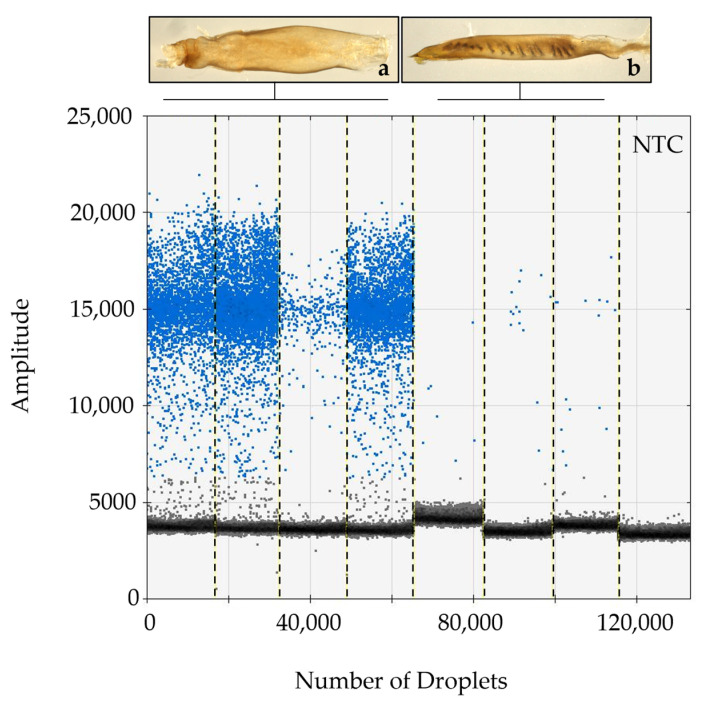
HzNV-2 is detectable at high levels in dissected sterile *H. zea* males compared to morphologically normal dissected *H. zea* males. Results from ddPCR using primer set P13 indicate the presence of HzNV-2 with positive droplets in blue and negative droplets in grey from agonadal *H. zea* males (**a**), typical *H. zea* males (**b**), and a no tissue control (NTC). Inset photographs from dissections made for this study illustrate the differences in male genital morphology between agonadal males (**a**) and typical males (**b**).

**Figure 2:**
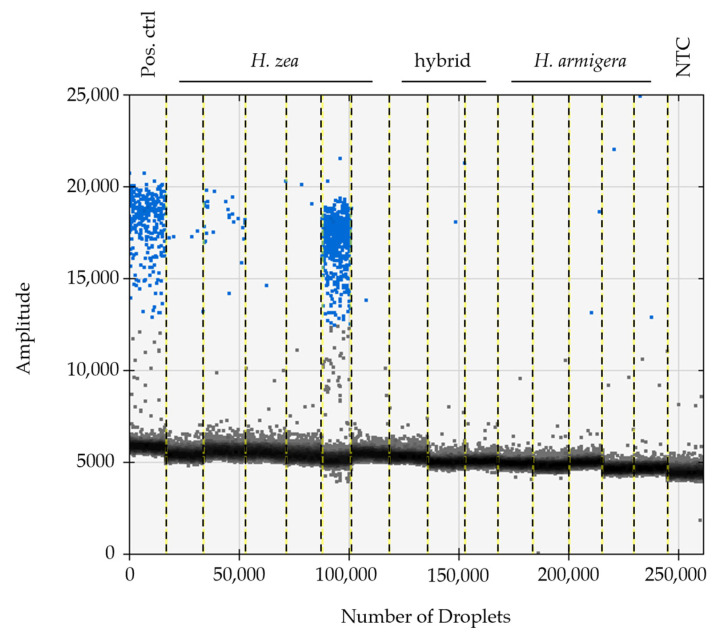
The ddPCR assay using the P13 primer set can detect varying concentrations of viral DNA present in specimens of *H. zea*. DNA from specimens of field-caught *H. zea* and *H. armigera*, and lab-reared hybrids used in the study were assayed using ddPCR to determine the presence or absence of viral DNA. The total DNA (host and virus) concentration is normalized to 0.1 ng/µL. Positive droplets are shown in blue, negative droplets are shown in grey.

In all instances where dissections were completed, the sterile agonadal individuals were visually aberrant, usually missing all cornuti in the vesica, and the vesica deformed or absent. Using the ddPCR assay, we were able to detect the presence of viral DNA in all agonadal samples collected in the USA with the P13 primer set (Table 1), indicating high levels of viral DNA in the agonadal samples. Some *H. zea* samples with typical genitalia also showed amplification in the form of a small number of positive droplets that were not present in the No Tissue Control (NTC) assays run with water instead of DNA (Figure 1). No HzNV-2 DNA was detected in *H. armigera* or hybrids (Figure 2). A set of 26 undissected individuals from across the USA were tested for HzNV-2 with ddPCR (primer set P13) of which eight (31%) had detectable levels of HzNV-2 DNA (Supplementary Table S1). Similarly, 29 bulk samples containing between three and 200 individuals (1014 individuals in total) from across the USA were processed with ddPCR with 17 (59%) containing detectable amounts HzNV-2 DNA (Supplementary Table S2). Among the bulk samples, those from Indiana were the only set that did not contain detectable levels of HzNV-2 whereas all samples from Colorado, Florida, and Texas contained detectable levels of virus with the remaining sample sets having a mixture of positive and negative samples.

### 3.2. Genomic Comparisons between HzNV-2, Host Species H. zea, and Closely Related Species H. armigera and C. virescens

The complete HzNV-2 genome was compared to whole genome contigs of *H. zea*, *H. armigera*, and *C. virescens* separately using BLAST searches (Table 2). The searches were performed against each genome separately to optimize results for recently endogenized genome segments between the virus and each lepidopteran species, and to minimize the number of hits to unrelated species. When DNA and protein annotations from viral ORFs agreed, matches were considered high quality representing probable recent endogenizations with functions potentially similar to those in the host genome. These searches revealed four ORFs shared between the HzNV-2 genome and all three individual lepidopteran genomes that could be considered recently acquired based on gene structure and sequence similarly. These results were found to have the lowest e-values across all searches. The four ORFs were annotated as ribonucleotide reductase (RNR), cytosolic serine hydroxymethyltransferase (cSHMT), proton-coupled folate transporter (PCFT), and thymidylate synthase (TS) based on both DNA and protein searches (Table 2; Supplementary Figure S1). These annotations were similar to those in Burand et al. [16] but additions to the GenBank database since 2012 have improved the specificity of annotations and the e-values of the results. Five other hits were found among the individual searches that had a 0.01 or lower e-value among at least one species. Of the remaining five hits associated with identifiable ORFs, annotations between DNA and protein searches were not as closely linked as those for the top four hits. The exception to this was ORF Hz2V091 (Table 2) that was inferred to be homologous with DNA and protein sequences from several bacterial species. ORF Hz2V091 was not annotated in Burand et al. [16].

### 3.3. Phylogenetic Analyses of HzNV-2 Genes of Recent Exogenous Origin

Because BLAST hits do not provide lineage-based gene comparisons, we also conducted several phylogenetic analyses using longer complete protein-coding sequences to assess gene origin and timing of acquisition in HzNV-2. When employing a taxon-constrained method, the genes cSHMT, PCFT, and TS all resolved with a lepidopteran clade across all three methods (BI, NJ, and parsimony). The RNR gene only resolved with the lepidopteran clade when using the Bayesian inference (BI) method (Supplementary Figure S2A). Regarding branching order, the ORF sequences from HzNV-2 resolved on an early diverging branch to the lepidopteran clade except for cSHMT which resolved late diverging and sister to *H. armigera* in the BI tree (PP = 0.88) and early diverging in the NJ tree (JK = 100) and parsimony tree (BS = 85; Supplementary Figure S2B). Resolution of the other insect lineages used here generally followed previous analyses (http://tolweb.org/Endopterygota/8243 accessed on 24 February 2021; Supplementary Figure S2C,D) for insects except for the placement of the lepidopteran clade in the PCFT tree where dipteran and hymenopteran clades are sister in the BI (PP = 0.87) and NJ methods (JK = 98) but not the parsimony method.

**Figure 3:**
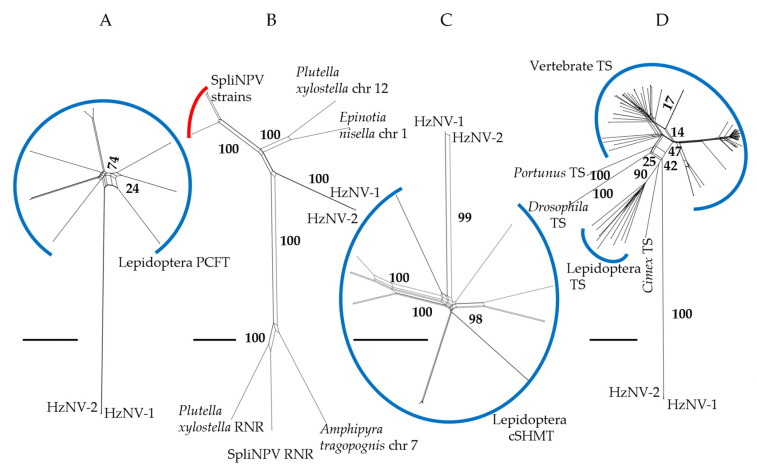
Splits networks from alignments of HzNV-2 ORFs with GenBank best hits using HzNV-2 ORFs (**A**) PCFT; (**B**) RNR; (**C**) cSHMT; and (**D**) TS. Numbers in networks are bootstrap values and a 0.1 scale bar is provided for each network for comparison at lower left. Blue arcs represent genes from a given metazoan lineage and the red arc indicates genes from a viral lineage. Star or branching patterns in the networks reflect a more even distribution of genetic distances between sequences whereas splits reflect clustering between groupings of sequences. See Figure S2 for the taxonomy constrained phylogenetic analyses.

When using a sequence similarity-only approach, splits networks for PCFT and cSHMT (Figure 3A,C) resolved viral ORFs with other lepidopteran genes with the same annotations. The cSHMT viral ORFs resolved on a branch similar in length to branches separating lepidopteran genes, while in the PCFT network the viral ORFs resolved on a long branch relative to the difference between lepidopteran genes. In the RNR splits network (Figure 3B) the viral ORFs resolved between clades containing lepidopteran genes and genes from *Spodoptera litura* Nucleopolyhedrovirus (SpliNPV) with branch lengths similar across the network. In the TS splits network the viral ORFs resolved on a long branch with other arthropod TS genes with splits separating this clade from a vertebrate TS clade (Figure 3B).

**Figure 4:**
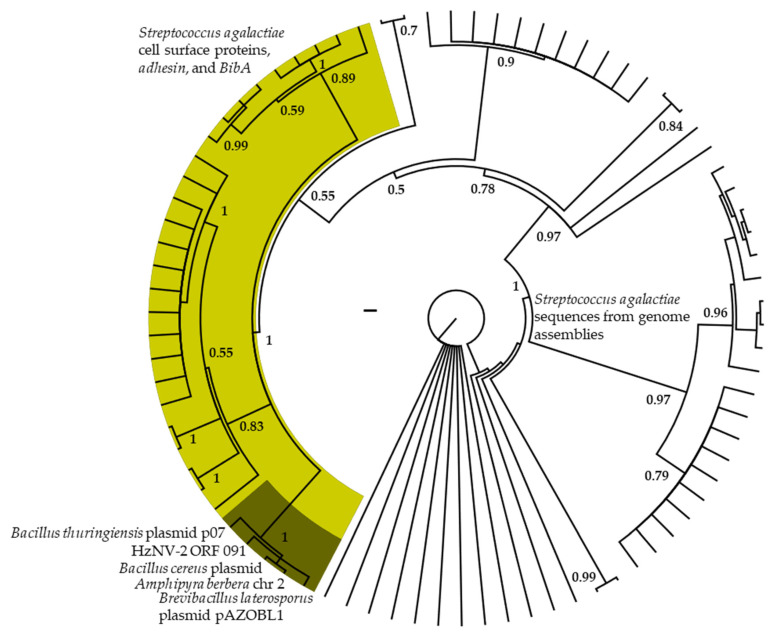
Phylogenetic analyses of the dr4 containing region of the HzNV-2 ORF Hz2V091 in an alignment with the best DNA matches from GenBank. The light-colored clade contains sequences annotated as cell surface proteins including *Adhesin* and *BibA*. The dark colored clade includes HzNV-2 ORF Hz2V091 and plasmid sequences from *Bacillus*, *Brevibacillus,* and the lepidoptera species *Amphipyra berbera*. All other terminals in the tree are from *Streptococcus agalactiae* genome assemblies. Posterior probability is shown for clade support and a 0.2 scale bar is shown near the center of the tree.

The phylogenetic analyses of ORF Hz2V091 (Figure 4) resolved this sequence in a well-supported clade (PP = 1) with sequences from plasmids of *Bacillus thuringiensis*, *B. cereus*, and *Brevibacillus laterosporus* as well as the noctuid species *Amphipyra berbera*. This clade was nested within a well-supported clade (PP = 1) of similar sequences from *Streptococcus agalactiae* with functional annotations for cell surface proteins such as *adhesin* and *BibA*. The alignment of ORF Hz2V091 to the best BLAST hits (Supplementary Figure S3A) indicates that the region of similarity between the *A. berbera* and many bacterial matches is centered on direct repeat 4 (dr4) near the 3′ end of the ORF. In addition, a region of similarity between a *Plasmodium* repeat motif and the 5′ end of ORF Hz2V091 was also matched. An uncharacterized gene in *H. zea* and *H. armigera* (mRNA XM_049842877) with a region similar to ORF Hz2V091 dr4 and bacterial sequences was also found in several recently produced *Helicoverpa* genomes (Supplementary Figure S3B).

### 3.4. Differences in Length and Nucleotide Content of cSHMT in HzNV-2, H. armigera, H. zea, and Hybrids

**Figure 5:**
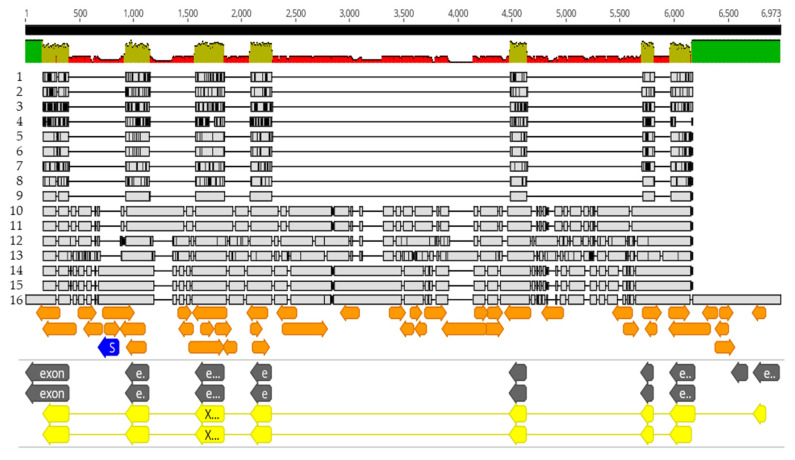
Alignment of cSHMT sequences. Below the alignment: orange tracks indicate alternative ORFs, gray tracks exons, yellow cds, and blue the SINE insert. The top gray and yellow tracks indicate isoform X2 and the bottom gray and yellow tracks indicate isoform X1. Sequences 1-9 are cds alignments from (1) *Culex quinquefasciatus*, (2) *Aedes albopictus*, (3) *Anopheles gambiae*, (4) HzNV-2, (5) *Bombyx mandarinia*, (6) *B. mori*, (7) *Trichoplusia ni*, (8) *Spodoptera litura*, and (9) *Helicoverpa armigera*. Sequences 10–16 are complete scaffold sequences from (10) *H. armigera* NW_018395393.1, (11) a HiRise assembly for *H. armigera*, (12) a HiRise assembly for *H. zea* x *H. armigera*, (13) a HiRise assembly for *H. zea* x *H. armigera* (14) a HiRise assembly for *H. zea*, (15) *H. zea* NFMG01027888.1, and (16) *H. zea* reference genome NC_061460.1.

**Figure 6:**
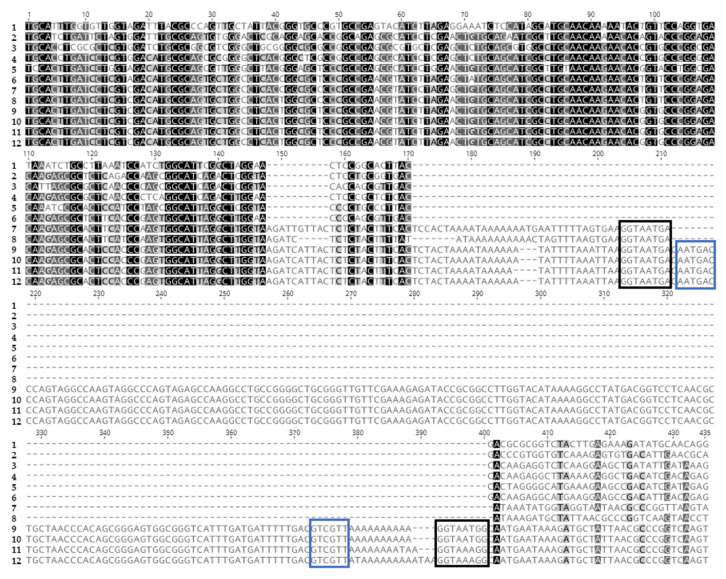
PCR-generated sequences from intron 7 of cSHMT aligned with sequences from GenBank. The sequences 7–12 were generated as part of this study except 11 which is from *Helicoverpa zea* NFMG01027888.1. The sequences are as follows: (1) *Anopheles gambiae*; (2) ORF Hz2V066; (3) *Bombyx mori*; (4) *Trichoplusia ni*; (5) *Spodoptera litura*; (6) *H. armigera*; (7) *H. zea* heterozygote light band; (8) *H. zea* × *H. armigera* lab reared light band; (9) *H. zea* heterozygote heavy band; (10) *H. zea* × *H. armigera* lab reared heavy band; (11) *H. zea*; and (12) *H. zea* field collected. Sequences from hybrids and heterozygotes were separated by gel electrophoresis, excised, and purified. Black boxes indicate TE flanking direct repeats and blue boxes indicate inverted repeats. *H. zea* sequence 11 was manually adjusted to align flanking direct repeats, the 5′ and 3′ ends of the alignment were trimmed to fit the figure.

PCR amplifications were conducted across multiple samples to confirm the presence or absence of a 200 bp insert inferred in intron 7 of the cSHMT gene in *H. zea* (Supplementary Figure S4). Both agarose gels and aligned Sanger sequence (GenBank accessions for sequences generated in this study OR609382-OR609386) results confirmed the presence of a 200 bp insert in intron 7 (Figure 5 and Figure 6). All lab-reared hybrids and several field-collected *H. zea* were found to have two prominent bands separated by about 200 bp when the PCR products were resolved on agarose gel. Among the hybrid samples, a presumably *H. armigera*-specific band shorter than the *H. zea* short band was evident especially in samples a/z 3 and a/z 4 which contain the shorter bands from both species. A faint intermediate band was present in some double-banded samples but was not assessed further at this time. Because some field-collected *H. zea* samples had similar banding patterns to known lab-reared hybrids, a real-time PCR assay was run to confirm whether these field-collected individuals were hybrids. None of the double banded *H. zea* were found to be hybrids when assessed with real-time PCR (Supplementary Figure S5). As such, the double-banded *H. zea* were considered heterozygous for the insert. This was further confirmed with HiRise assembled genome data from a hybrid that did not contain the insert (Figure 5). When the insert sequence was BLAST-searched against the reference *H. zea* genome, homologous sequences were found in 10,963 separate locations in the genome although many were shorter than the query sequence (Supplementary Figure S6). The contribution of this insert to the total length of each chromosome was proportionally higher among the shorter chromosomes (Supplementary Figure S7). Because cytochrome P450 genes are known to be central to mediating xenobiotic responses including viral infection in insects and have similar inserts as those found in cSHMT, we specifically examined these genes for such inserts. We found 13 cytochrome P450 genes with similar inserts from seven chromosomes mainly in the introns but with several spanning intron exon boundaries as well as inserts closely adjacent (less than 200 bp) to a gene (Table 3). Sequence similarity between the insert found in *H. zea* cSHMT and those described from *H. armigera* [72,73] suggests that this insert is a type of transposable element (TE) known as a short interspersed nuclear element (SINE). Specifically, the insert contains the imperfect direct repeats 5′-GGTAATGA-3′ at the 5′ and 5′-GGTAATGG-3′ at the 3′ ends of the insert and within each of the direct repeats are inverted repeats 5′-AATGAC-3′ and 5′-GTCGTT-3′ (Figure 6). Such a pattern of nucleotide repeat flanking is consistent with TE insertion [74,75].

## 4. Discussion

The dissected agonadal specimens examined in this study (consistent with the description of *H. stombleri*) produced many positive droplets from HzNV-2 DNA when amplified using ddPCR (Figure 1). The agonadal samples with a lower number of droplets still constitute a much higher number of positive droplets than the asymptomatic individuals. The lower number of droplets in this sample might be the result of several factors including differences in tissue collection, DNA extraction efficiency, sample preservation, and viral load. The three dissected individuals consistent with typical *H. zea*, which did not have malformed agonadal genitalia, had very few to no positive droplets. The presence of a small number of droplets sharing a consistent amplitude (RFU) with a positive result from asymptomatic samples has several possible explanations. Since this assay is not intended for diagnosis of the condition, we did not determine a false positive rate. A small number of false positives is considered standard in most ddPCR assays (e.g., [76,77]). Another explanation, which is likely due to the consistency of results for individuals between runs and the lack of false positives in NTC reactions, is that these are asymptomatic (or possibly latent) carriers of HzNV-2 with lower virus titers [20,78]. In any case, a clear difference in the number of positive droplets can be seen between the *H. stombleri*/agonadal forms (216–4580 copies of target per µL total DNA) and the typical *H. zea* samples (2.1–8.3 copies of target per µL total DNA; Figure 1). The lack of positive ddPCR results for *H. armigera* and hybrids indicates that HzNV-2 was not present in these specimens (Figure 2).

A set of undissected individuals and bulk samples were run using the ddPCR assay to estimate the prevalence of HzNV-2 in *H. zea* (Figure 2; Supplementary Tables S1 and S2). Among the individual samples no obvious geographic pattern of prevalence was observed; however, two samples (sample numbers 2330 and 3519) from Puerto Rico collected in different years were found to have very high copy numbers of HzNV-2 present (5520 and 2821 positive droplets). Like the individual sampling, our bulk sampling found the presence of HzNV-2 in nearly all locations except for Indiana which had small bulk sample sizes possibly explaining why no virus was detected there. Our sampling results included a broader geographic area and larger number of samples than Lupiani et al. [12], yet produced similar results, further supporting their assertion that HzNV-2 is an endemic virus to *H. zea* and not recently released from a small area of endemism in Mississippi as had been previously thought. The endemism of HzNV-2 is further confirmed when considering the frequent finds of sterile *H. zea* in South America and other parts the species range [24] are most likely the result of infection. Our sampling, together with past observations, make the case for the need to conduct intensive screening studies for HzNV-2 such that fine scale patterns associated with geographic location, ecological setting, and phenology can be related to prevalence and viral load in *H. zea* populations. Such studies could be applied to understanding how viral infection affects *H. zea* population dynamics and, in turn, crop health. Our results show that ddPCR is an excellent tool for conducting such studies. Pairing epidemiological ddPCR studies with high-throughput sequencing of viral genomes could allow for real-time monitoring of gene introductions from host and other sources, including transient TEs and short non-coding fragments.

Because of the extensive changes to genitalic morphology caused by HzNV-2 infection [17,19] and the importance of genitalia for species identification [79,80], it is not surprising that a species-level taxon designation (i.e., *H. stombleri*) was proposed to account for such observed changes. Given the fact that numerous other sexually transmitted viruses, and viruses in general, can alter the morphology of insect genitalia [14] and other morphological characters [81], it is possible that other insect taxonomic designations have been based on traits altered due to viral infection. This might be especially problematic in cases where the resulting morphological change is not as significant as that caused by HzNV-2 or, in the extreme case, has a selective advantage, as in the domesticated viruses in some ichneumonid wasp lineages [9,82]. Insect species descriptions based on genitalia characters that disagree with other data (e.g., gene trees) may warrant further review considering viral infection as a source of morphological change. Such studies might help to clarify taxonomic inconsistencies among hosts as well as be valuable in discovering new species of viruses and novel host–pathogen interactions.

In our dataset, four genes were found to be very similar in genomic comparisons of HzNV-2 with *H. zea*, *H. armigera*, and another common heliothine pest *C. virescens* (Table 2). In these instances, timing of gene acquisition is reflected in the BLAST results and phylogenetic analyses of the complete ORF sequences. For the RNR BLASTn search, the best hit was to *Spodoptera littoralis* nucleopolyhedrovirus (SpliNPV) and the best PSI-BLASTp was to an RNR from *Hyposmocoma kahamanoa* (Lepidoptera: Cosmopterigidae), while the taxonomically constrained phylogenetic analyses of the complete ORF sequences had HzNV-2 RNR resolving with the other lepidopteran species only in the Bayesian method and branching in a basal position from the outgroup in the other two methods (Supplementary Figure S2). The sequence similarity approach resolved the HzNV-2 RNR ORF with other viral and lepidopteran RNR genes (Figure 3B). This pattern of matches to both lepidopteran and viral sequences may suggest a more ancient acquisition of this gene by an ancestral nudivirus/baculovirus with subsequent sorting to extant viral lineages such as SpliNPV and HzNV-2. Burand et al. [16] identified 20 other ORFs (including a second copy of RNR) from HzNV-2 that were similar to ORFs from other nudiviruses and baculoviruses which may reflect a shared history of a single acquisition event for each ORF among related Nudiviridae species. Alternatively, the shared presence of RNR in separate viral lineages may be the result gene exchange between virus species during a simultaneous infection event which has been noted previously in other viruses [83,84]. The acquisition of RNR by other dsDNA viruses appears to have occurred multiple times as is evident from the presence of different RNR classes in distinct viral lineages similar to non-viral RNRs [85]. Given this frequent uptake of RNR by dsDNA viruses the shared copies in SpliNPV and HzNV-2 may also be explained by separate convergent acquisitions. The acquisition of RNR by viruses is often associated with increasing viral autonomy through the synthesis of deoxyribonucleotides needed for DNA replication of the viral genome independent of host cellular machinery [86,87]. Alternative functions for RNR subunits such as blocking the host inflammatory response and innate immune signaling have been described for viral RNRs, which corresponds with the loss of original enzymatic function of the acquired gene [88].

The PCFT and TS genes resolved in the same position across all three taxon-constrained phylogenetic methods with relatively high support values in all instances. The acquisition of TS in viral genomes is often associated with RNR as it produces a necessary enzyme for the synthesis of the DNA precursor 2′-deoxythymidine-5′-monophosphate [89,90]. The RNR and TS ORFs in HzNV-2, as well as others such as ORF Hz2V067 (Deoxynucleotide kinase) and ORF Hz2V069 (dUTPase), are likely involved with viral DNA replication but alternative functions could have evolved for these or similar genes (such as a second copy of RNR, ORF Hz2V065) as has occurred in herpes simplex virus [91]. The presence of PCFT in the HzNV-2 genome could be functionally associated with cSHMT (potentially both host and viral forms) as they both function in eukaryotic folate metabolism [92,93] and might similarly shuttle/regulate such molecules between virus and host in this instance as well.

The cSHMT ORF from HzNV-2 matches with very high confidence to *H. armigera* cSHMT sequences in both the BLASTn and PSI-BLASTp searches (Table 1). Similarly, the complete ORF phylogenetic analyses using a taxonomy-constrained alignment placed the HzNV-2 sequence with the lepidopteran species in an early diverging position by NJ and parsimony, or sister to *H. armigera* in the Bayesian method (Supplementary Figure S2). For both RNR and cSHMT the similarity-based methods placed the HzNV-2 sequence in a more distant relationship than the model-based Bayesian method, but the placements were consistent in the degree to which the HzNV-2 sequences were similar to other lepidopteran sequences. In the splits network, the HzNV-2 ORF resolved with other lepidopteran cSHMT genes on a relatively short branch with few internal splits suggesting minimal evolution between host and viral genes perhaps as a result of recent acquisition (Figure 3C). High levels of sequence similarity are also reflected in the BLAST hits to the nucleotide and protein databases. From these results it is reasonable to infer that cSHMT may be the most recently acquired host gene. The recent acquisition of cSHMT from a lepidopteran host species, and the limited host specificity of HzNV-2, is consistent with gene acquisition as a speciation/adaptive event, as seen in similar viruses [40].

One-carbon units such as those produced by cSHMT are essential to many cellular pathways. For instance, cSHMT plays an important role in processes such as neurotransmitter synthesis, lipid and protein production, maintenance of cellular redox status, folate metabolism, and methylation [94,95]. In insects, cSHMT most likely functions similarly to that of other eukaryotes [96] but may also play a role in virus inhibition as has been shown for cytosine methyltransferases in *Aedes aegypti* controlled via *Wolbachia* [97]. Similarly, the cSHMT copy in the HzNV-2 genome (which is unique among xenologous viral genes) may have alternative functions beyond DNA replication including escape from host defense as with RNR in some herpes viruses [88]. The presence of multiple nested putative ORFs in viral and lepidopteran cSHMT (especially nested intronic ORFs in *H. zea* and *H. armigera*) may also point to additional uncharacterized functions and interactions for this gene (Figure 5; [98]).

From alignments of publicly available sequence data, HiRise assembled genomes, and PCR generated sequences from *H. zea*, and *H. zea* x *H. armigera* hybrids, the cSHMT gene of *H. zea* appears to have undergone several changes (mainly to intronic regions) compared to the sister species *H. armigera* (Figure 5 and Figure 6). Given the presence of cSHMT in HzNV-2, the structural changes in *H. zea* cSHMT may affect host–virus interaction. We focused on the SINE found in intron 7, as homologous sequences were found throughout the *H. zea* genome in association with functional genes such as cytochrome P450. The cytochrome P450 family of enzymes are well known in the metabolism of xenobiotics [99] and have thus been much studied for their role in producing resistance to various insecticidal compounds [100,101]. In addition to metabolizing various xenobiotic compounds, cytochrome P450 enzymes have also been described in several types of pathogen defense responses [102,103]. The insertion of TEs in or near *H. armigera* cytochrome P450 genes has been linked to increased resistance to insecticidal compounds [72]. While TE insertion into genes can result in a loss of function, they have also been noted in providing functional advantages such as linking genes from distant regions of the genome through coordinated transcription in response to an external agent [104]. As such, could the potential linking of *H. zea* cSHMT to different cytochrome P450 genes (as well as many other genes) be related to the interaction of host and viral copies of cSHMT? Examples of cytochrome P450 genes responding to viral infection have been observed in *H. zea* to HzSNPV (*H. zea* single-nucleocapsid nucleopolyhedrovirus) [105], and *Trichoplusia ni* to *Autographa californica* multiple nucleopolyhedrovirus [106] as well as in more distant lineages such as *Aedes aegypti* to Dengue virus infection [103]. Viral infections have also been noted to increase the abundance of host TE transcripts in *Drosophila* [107]. That said, TE insertion may have some negative impacts on proper functioning of cSHMT, although mitochondrial copies of SHMT have been shown to be able to compensate in instances of loss of function in cSHMT in mammalian models [108,109]. To this point, it should be noted that all *H. zea* that were heterozygous for the TE in intron 7 of cSHMT were not found to contain HzNV-2 DNA (Table S1). As our sample sizes were small, more follow-up work should be conducted to understand how TE-bearing cSHMT copies and viral infection are correlated. Lastly our observation of hybrid gel banding patterns (Supplementary Figure S4) as well as sequence data (Figure 6) show that the TE in cSHMT is inherited in most hybrids (especially given the high rates of TE homozygosity in *H. zea*) which may provide novel adaptive links between genes which are not present in either of the parents, potentially exacerbating the spread of coadapted hybrids in the Americas [21]. The different arrangements of *H. zea* and *H. armigera* cSHMT (especially regarding intron sequences) will provide a useful variable locus for the development of species- and hybrid-level markers for use in screening projects. The presence of cSHMT in HzNV-2 is unique among viruses studied thus far and may provide an exceptional system (virus–host interaction and evolution) for understanding how this essential gene functions and novel methods for blocking or altering this gene in the development of antifolate-like medications [90,110,111,112] or the development of folate-disruption-based sterility techniques for pest insect control [113].

When the Hz2V091 ORF was BLASTn searched, the region of greatest similarity was associated with the direct repeat (dr) 4 identified in Burand et al. [16] to numerous bacterial sequences (Supplementary Figures S1 and S3). Such repeats are known to be involved with creating binding recognition sites in translated proteins associated with bacterial cell surface interactions with host cells [114,115]. In addition, binding proteins are known to evolve via modular assemblage/recombination outside of the dr sites [116,117,118]. This is consistent with the bacterial dr-containing gene in *Helicoverpa* made up of a 5′ region with matches to noctuid DNA, the dr region similar to HzNV-2 and bacterial motifs, and a region 3′ of the dr matching to several noctuid species (Supplementary Figure S3). This too appears to be the case in Hz2V091, with a 5′ region matching to repeats from *Plasmodium* and the dr4 region matching to bacterial sequences. Some of the outstanding questions related to this genomic region are: (1) How and when did this gene or motif (drs) transfer to or evolve in *H. zea* or HzNV-2 and what function does it have? (2) Did HzNV-2 act as an intermediary from bacteria to *H. zea*? (3) Was the dr-containing gene in *H. zea* the source of Hz2V091 with recombination in HzNV-2 thereafter to attain the current ORF sequence? (4) Did virus and host acquire the same gene motifs through separate unrelated events? More work is needed to answer these questions but a functional role for the dr-containing gene in *Helicoverpa* is reasonable based on similar genes from other insects [119] and the presence of the dr4-like sequence in the cds of gene mRNA XM_049842877. Additionally, the dr4 match in *Helicoverpa* genomes is not arrayed throughout the genome or paired with other similar drs and thus not convergent to viral or bacterial copies through chance paralogous duplication and mutation.

The acquisition of genes from bacteria that co-occur in the environment or coinfect host cells appears to be commonplace among large dsDNA viruses [84,120,121]. From analysis of several large dsDNA virus genomes, most genes acquired from bacteria are related to DNA replication and repair, or are membrane proteins [120]. Only two other HzNV-2 ORFs (Hz2V099; *Psychromonas ingrahamii*, esterase/lipase and Hz2V110; *Trichomonas vaginalis*, protein kinase) were found to contain sequences matching to other microbes in the Burand et al. [16] annotation. Most of the BLAST hits for ORF Hz2V091 in our comparisons were for cell surface proteins suggesting a possible cell-surface recognition/manipulation function for this gene [122].

The cooccurrence of HzNV-2 and bacteria such as *Brevibacillus laterosporus* and *Bacillus thuringiensis* in the insect host [123] provide suitable conditions for HzNV-2 to acquire bacterial genes for novel functions that may influence fitness. While HzNV-2 and similar viruses have been shown to acquire bacterial genes, the degree to which bacteria may be involved in the life cycle of these viruses, in roles such as secondary hosts, vectors, or agents of coinfection increasing pathogenicity (e.g., [124]), has not been thoroughly studied. The ORFs with motifs of bacterial origin in HzNV-2 are clustered closely in the genome, a pattern found in other large dsDNA viruses [120]. The genomic region where Hz2V091 and the 5′ and 3′ flanking ORFs (ORFs Hz2V090 and Hz2V092 with regions similar to *Acinetobactor soli*, e-value = 3 × 10^−53^ and *A. junii*, e-value = 1 × 10^−42^, respectively, as attained from BLASTn searches) are located in the HzNV-2 genome is absent in the closely related HzNV-1 [16]. This suggests that this cluster of three ORFs may have been recently acquired by HzNV-2 or lost by HzNV-1 and by extension could be an important source of functional differences. Additionally, this region is enriched in direct repeats [16], with the Hz2V090 ORF possessing a protein motif from a DNA intra-strand crosslink recognition protein (e-value = 8 × 10^−154^ top hit when Hz2V090 is BLASTp searched against the nr database) which suggests that this area may be a recombination and exogenous DNA uptake hotspot [125,126,127]. The acquisition not only of host genetic material but of genetic material from bacteria and other microbes may be critical in gaining novel functions, adapting to new hosts, altering viral life cycles, broadening vector dynamics, and ultimately resulting in functional differences between HzNV strains as well as in other large dsDNA viruses [84,120,121]. Because HzNV-2 appears able to acquire bacterial genes (or parts of bacterial genes), these types of viruses might also be involved with symbiont-induced insect speciation through the manipulation of microbial symbionts and/or host–microbe interactions [28].

Viral infection alone is unlikely to result in speciation of the host. However, long-term coexistence between virus and host can affect populations coevolving with viral pressure differently by providing an ‘environment’ more favorable to high mutation rates [128,129] versus populations without viral pressure, potentially increasing divergence rates. Rapid evolution of host response and immunity loci may not result in reproductive isolation of populations with viral pressure but could result in local extinction of infected populations without immunity and, in turn, accelerate allopatric speciation [130,131]. Viruses, in rare events, can also have a more direct effect on divergence by increasing the fitness of hosts when genetic rearrangements (including viral integration) in response to foreign DNA result in a gain of novel function [9,29,38,132]. This may allow an insect population to occupy a new niche and could help drive ecological speciation. A pattern of differentiation is evident when comparing *H. zea* and *H. armigera* where divergence in the cSHMT gene was noted between these two species. Given this pattern between the two *Helicoverpa* species, HzNV-2 may have evolved to infect *H. zea* after or as part of the divergence of *H. armigera* from *H. zea*. If this is the case, then HzNV-2 may have evolved to infect *H. zea* in the last 1.5 my based on whole genome divergence estimates between *H. zea* and *H. armigera* [133]. The closely related baculoviruses HaSNPV (*H. armigera* SNPV) and HzSNPV provide a useful comparison to HzNV-2 evolution given that speciation in the two baculovirus species followed host speciation [134]. If assuming a similar time since divergence between the two *Helicoverpa* SNPVs and HzNV-2 from a yet undetermined sister lineage, the rate of host-gene acquisition since divergence varies, with one acquisition in HzSNPV, to three in HaSNPV to four or more in HzNV-2 when using *H. armigera* wgs contigs as a basis for comparison (Supplementary Figure S8). Viral prevalence also seems to vary between viral lineages with rates of 31% in individual samples and 58% found in bulk samples for HzNV-2 from this study (Supplementary Tables S1 and S2), while infection rates of only 13% were noted from a field study of wildtype HaSNPV [135]. These differences could be related to the lifecycles of these viruses where latent infections have been noted in HzNV-2 [78] and high rates of mortality are known from *Helicoverpa* SNPVs. As with many questions in evolution, the determination of event order becomes an important part of linking pattern to process. For instance, did the ancestors of HzNV-2 acquire numerous essential host genes resulting in longer viral persistence in the host (and as a result, higher rates of prevalence), or did some other factor enable longer viral persistence in the host and increase the opportunity for host-gene acquisitions? Given the pronounced differences and shared ancestry between baculoviruses and nudiviruses, these lineages provide a superb comparative study system for understanding viral evolution.

Insect-virus infections should be characterized in greater detail, including HzNV-2 with *Helicoverpa,* to understand how these viruses might mediate speciation events and how viral, microbial, and host genes interact at the molecular level. Among animals, the insects (Hexapoda) contain the greatest species diversity [136], and if the number of viruses in each insect species is similar to that of other animal lineages [137,138], the number of insect viruses could greatly exceed 50 million (=1 million described insect species × 50 viruses/insect species). As such, the greatest number of animal viruses and by extension novel molecular interactions, some of which could have applications for biotechnology, are likely to be found among insects. Further studies of viruses should be encouraged to gain a better understanding of how they have influenced insect evolution and, in turn, the biosphere.

## Figures and Tables

**Table 1:** Primers used in this study.

Name	Description	Sequence	Tm (°C)	Source
P4-I	HzNV-2 detection	5′-GCACGATTCGTAATGTTC-3′	51.8 *	[12]
P4-II	HzNV-2 detection	5′-GCACACCTATCAATCACC-3′	52.8 *	[12]
P13-I	HzNV-2 detection	5′-TCGATGCCGTAATACC-3′	49.7 *	[12]
P13-II	HzNV-2 detection	5′-GTCGCTGAATCAAGTCTG-3′	53.2 *	[12]
Hz_SHMT_1738F	cSHMT TE insert intron 7	5′-CCAGCGCCTCTGCAAAGG-3′	61.4	This Study
Hza_SHMT_24R	cSHMT TE insert intron 7	5′-TAAATGTTAAGCTGTTRTCCTCT-3′	53.2–55.9	This Study

* Tm values were recalculated for these primers using the same methods as for the PCR primers designed in this study.

**Table 2:** Annotations and e-values for genomic comparisons and database searches. Values in parenthesis after the best DNA hits are e-value/% query coverage/% identity from the best hit.

Location in HzNV2 Genome	Annotation from Similar Sequences	*H. zea*	*H. armigera*	*C. virescens*	Best DNA Hit	Best Protein Hit	Putative Viral Function	Burand et al. Annotation
ORF Hz2V047 (96009-98822)	Ribonucleotide reductase	1 × 10^−30^	6 × 10^−34^	8 × 10^−21^	*Spodoptera littoralis* nucleopolyhedrovirus isolate SpliNPV-Tun2 (2 × 10^−130^/66/67.1)	Ribonucleoside-diphosphate reductase large subunit, *Hyposmocoma kahamanoa* (0.0)	Nucelotide anabolism, inhibit host cell signalling	Ribonuclease reductase
ORF Hz2V066 (127832-126510)	Serine hydroxymethyltransferase	7 × 10^−27^	5 × 10^−22^	6 × 10^−23^	Serine hydroxymethyltransferase, cytosolic transcript variant × 2, *Helicoverpa armigera* (5 × 10^−113^/88/67.2)	Serine hydroxymethyltransferase, cytosolic isoform × 1, *Helicoverpa armigera* (0.0)	Nucelotide anabolism, one-carbon metabolism	Serine hydroxymethyltransferase
ORF Hz2V023 (49772-51307)	Proton-coupled folate transporter	2 × 10^−21^	2 × 10^−20^	3 × 10^−20^	Proton-coupled folate transporter, *Helicoverpa armigera* (3.0E-166/81/70.8)	Proton-coupled folate transporter, *Helicoverpa armigera* (0.0)	Folate transport	Membrane transporter
ORF Hz2V035 (69749-70621)	Thymidylate synthase	1 × 10^−18^	3 × 10^−18^	5 × 10^−17^	Thymidylate synthase, *Pectinophora gossypiella* (3 × 10^−90^/90/70.3)	Thymidylate synthase, *Manduca sexta* (7 × 10^−170^)	Thymidine biosynthesis	Thymidylate synthesis
ORF Hz2V002 (7183-13047) hit associated with nested ORF 12222-11431	unknown	2 × 10^−14^	1 × 10^−14^	no match	Chromosome 19, *Perizoma flavofasciatum* (2 × 10^−34^/60/74.6)	transcriptional regulatory protein AlgP, partial *Biomphalaria glabrata* (0.036)	unknown	Unidentified
ORF Hz2V098 (195699-199133)	unknown	7 × 10^−8^	1 × 10^−9^	no match	Chromsome7, *Amphipyra tragopoginis* (5 × 10^−81^/21/70)	Hypothetical protein Cantr_06524, *Candida viswanathii* (3 × 10^−4^)	unknown	Unidentified
ORF Hz2V091 (177136-178524)	Cell surface protein (bacterial origin)	1 × 10^−5^	3 × 10^−12^	no match	HD-771 plasmid p07, *Bacillus thuringiensis* (4 × 10^−34^/21/75.9)	Hypothetical protein BTXL6_27630, *Bacillus thuringiensis* (5 × 10^−23^)	Cell surface interaction	Unidentified
Between ORF Hz2V083 (158080-158743) and ORF Hz2V084 (163557-159694)	unknown	1 × 10^−6^	6 × 10^−9^	no match	Chromosome 6, *Harpalus rufipes* (1 × 10^−21^/12/68)	no match	unknown	Unidentified
ORF Hz2V062 (120105-118786)	Occlusion-dervived virus envelop protein e56	6 × 10^−4^	1 × 10^−5^	no match	Chromosome 14, *Lumbricus terrestris* (1 × 10^−7^/9/84.6)	PIF-5a, *Tipula oleracea* nudivirus (9 × 10^−38^)	Viral envelope synthesis	Unidentified

**Table 3:** All cytochrome p450 genes in the *H. zea* reference genome with detectable TE sequences similar to those found in intron 7 of cSHMT.

Chr	Length (bp)	% Identity	e-Value	5’ Position	Annotation from Reference Genome	Genic Location	Gene #
1	63	89.71	2 × 10^−17^	10,131,593	cytochrome p450 4C1-like	intron 7 of 7	1
5	183	73.30	4 × 10^−32^	13,156,359	138 bp from cytochrome p450 4C1-like	UTR	2
5	62	91.05	5 × 10^−18^	13,139,843	cytochrome p450 4C1-like isoform x1	intron 3 of 9	2
5	58	84.75	9 × 10^−09^	13,139,943	cytochrome p450 4C1-like isoform x1	intron 3 of 9 into exon 4 of 10	2
9	33	84.21	0.029	1,586,828	cytochrome p450 9E2-like	intron 2 of 9	3
9	83	94.05	7 × 10^−29^	1,590,440	cytochrome p450 9E2-like	intron 6 of 9	3
9	31	87.50	0.029	1,590,561	cytochrome p450 9E2-like	intron 6 of 9	3
15	170	73.14	2 × 10^−29^	11,280,289	cytochrome p450 6K1-like	intron 7 of 8	4
19	73	83.33	5 × 10^−18^	4,236,290	cytochrome p450 4G15-like	intron 5 of 10	5
19	168	75.15	9 × 10^−34^	4,240,146	119 bp from cytochrome p450 4G15-like	UTR	5
19	50	86.54	1 × 10^−6^	4,250,534	cytochrome p450 4C1-like	intron 4 of 10	6
23	44	85.71	4 × 10^−7^	7,407,358	cytochrome p450 4V2-like	intron 4 of 9	7
23	171	71.75	6 × 10-^24^	7,426,917	cytochrome p450 4C1-like	intron 3 of 9 into exon 4 of 10	8
23	41	85.71	7 × 10^−4^	7,447,063	cytochrome p450 4C3-like isoform x1	intron 2 of 10	9
23	63	86.77	9 × 10^−15^	7,447,155	cytochrome p450 4C3-like isoform x1	intron 2 of 10	9
23	45	82.00	7 × 10^−4^	9,722,754	cytochrome p450 4C1-like	intron 7 of 9	10
23	44	85.71	4 × 10^−7^	9,722,850	cytochrome p450 4C1-like	intron 7 of 9	10
23	82	87.06	1 × 10^−19^	9,783,243	cytochrome p450 4V2-like	intron 7 of 10	11
23	78	87.34	2 × 10^−18^	9,820,391	cytochrome p450 4C1-like	intron 2 of 9	12
30	70	97.00	5 × 10^−18^	2,071,964	cytochrome p450 307A1	intron 1 of 1	13

## Data Availability

All sequence data can be accessed through NCBI GenBank at the accession numbers OR609382-OR609386 for the Sanger data and under BioProject ID PRJNA1020878 for the genomic data. All other data are available by request, please contact Luke Tembrock (tembrock@colostate.edu).

## Supplementary Materials

The following supporting information can be downloaded at: https://www.mdpi.com/article/10.3390/insects14100797/s1. Table S1, Individual samples tested for presence of HzNV-2 with ddPCR. Samples withGenBank accession numbers were Sanger sequenced; Table S2, ddPCR results from bulk samples tested for prevalence of HzNV-2; Table S3, Results of model tests for each gene cds alignment used in the DNA-based phylogenetic analysis; Figure S1, Translated HzNV-2 ORFs inferred to be recent host acquisitions BLASTP searched against the clustered nr database; Figure S2, Phylogenetic analyses of sequences from A) RNR; B) cSHMT; C) PCFT; and D) TS. Figure S3, Best sequence matches mapped to A) HzNV-2 ORF Hz2V091 and B) an H. zea gene of unknown function with a central region similar to HzNV-2 dr4 in ORF Hz2V091; Figure S4, Agarose gel (1%) of PCR products spanning the TE insert in intron 7 of cSHMT from H. zea, and lab-reared hybrids. HzNV-2 test results on these samples can be found in Supplementary Table S1. *Refers to samples that were Sanger sequenced; accessions given in Table S1; Figure S5, The cSHMT Intron 7 TE-inserted specimen with gel banding pattern similar to H. zea x H.armigera lab hybrid specimens but is identified as H. zea using the validated real-time PCR method from Gilligan et al. (2015); Figure S6, The size distribution of TEs with similar sequence identities (cutoff e-value < 0.01) to those found in P450 genes and cSHMT intron 7 across a subset of chromosomes; Figure S7, The proportion of each H. zea chromosome made up of TEs with similar sequence identities (cutoff e-value < 0.01) to those found in P450 genes and cSHMT intron 7. Chromosomes ordered largest (15.51 Mb chr 1) to smallest (6.32 Mb chr 30) with the Z chromosome at 18.81 Mb listed last; Figure S8, Inferred recent host DNA acquisitions mapped to baculoviruses HaSNPV (blue), HzSNPV (green), and nudivirus HzNV-2 (orange). Arrows indicate inferred host DNA acquisitions since H. armigera and H. zea divergence (for the baculoviruses only as the sister species of HzNV-2 has not been determined). All searches used complete viral genomes as queries to wgs H. armigera taxid 29058. Viral genomes not to scale.
