# Supplementary material for: Viral Prevalence and Genomic Xenology in the Coevolution of HzNV-2 (Nudiviridae) with Host Helicoverpa zea (Lepidoptera: Noctuidae)
Source: Insects. 2023 Sep 30;14(10):797. doi: 10.3390/insects14100797 (PMC10607169; doi:10.3390/insects14100797)
Supplement: Supplementary file 1 [file insects-14-00797-s001.zip › insects-2503422-supplementary.pdf]

Supplementary Table S1 Individual samples tested for presence of HzNV-2 with ddPCR. Samples with GenBank accession numbers were Sanger sequenced.

Specimen ID	Species	date collected	Life Stage	Origin	Positive Droplets	Total Droplets	Concentration copies/uL of PCR	HzNV-2 presence/ absence	Fig. 1 column	Fig. 2 column	heavy band w/TE	light band	GenBank ID
123	Ha	3 Oct 2013	adult	Valencia, Spain	0	16334	0	0	n/a	11	n/a	n/a	n/a
141	Ha	Dec 2013	adult	CSIRO lab colony, Australia	0	16071	0	0	n/a	12	n/a	n/a	n/a
144	Ha	Dec 2014	adult	CSIRO lab colony, Australia	2	15211	0.15	0	n/a	13	n/a	n/a	n/a
269	Ha	5 Nov 2015	adult	Zambesiaca, South Africa	1	14581	0.08	0	n/a	14	n/a	n/a	n/a
343	Ha	Dec 2014	adult	CSIRO lab colony, Australia	2	15175	0.16	0	n/a	15	n/a	n/a	n/a
171	Hz	16 July 2013	adult	Yuma County, Colorado, USA	9	16128	0.66	1	7	n/a	n/a	n/a	n/a
351	Hz	25 July 2014	adult	Alamosa County, Colorado, USA	1	16373	0.07	0	n/a	n/a	1	0	n/a
352	Hz	25 July 2014	adult	Alamosa County, Colorado, USA	1	17302	0.07	0	n/a	n/a	1	0	n/a
353	Hz	25 July 2014	adult	Alamosa County, Colorado, USA	0	17651	0	0	n/a	n/a	1	0	n/a
355	Hz	25 July 2014	adult	Alamosa County, Colorado, USA	2	17937	0.13	0	n/a	n/a	1	0	n/a
464	Hz	20 Oct 2014	adult	McClean County, Kentucky, USA	668	18730	42.7	1	n/a	n/a	1	0	n/a
493	Hz	2014	larvae	LSU Ag Center, MaconRidge, Louisiana, USA	0	18962	0	0	8	n/a	1	0	Acc#
494	Hz	2014	adult	LSU Ag Center, MaconRidge, Louisiana, USA	0	17319	0	0	7	n/a	1	1	Acc#, #
495	Hz	2014	adult	LSU Ag Center, MaconRidge, Louisiana, USA	2	18046	0.13	0	n/a	n/a	1	0	n/a
1012	Hz	11 Aug 2015	adult	Shelby County, Tennessee, USA	12	16977	0.83	1	6	n/a	n/a	n/a	n/a
1111	Hz	18 Aug 2015	adult	Miami-Dade County, Florida, USA	5	17261	0.34	1	n/a	2	n/a	n/a	n/a
1116	Hz	18 Aug 2015	adult	Miami-Dade County, Florida, USA	5	19061	0.31	0	n/a	n/a	1	0	n/a
1155	Hz	18 Aug 2015	adult	Manatee County, Florida, USA	1	17451	0.07	0	n/a	n/a	1	1	n/a
1158	Hz	19 Aug 2015	adult	Manatee County, Florida, USA	27	18980	1.7	1	n/a	3	n/a	n/a	n/a
1751	Hz	1 Sep 2015	adult	Penobscot County, Maine, USA	36	18707	2.3	1	n/a	n/a	1	0	n/a
1877	Hz	25 Sep 2015	adult	Larimer County, Colorado, USA	5018	15560	458	1	2	1	n/a	n/a	n/a
2191	Hz	27 Oct 2015	adult	Guanica, Puerto Rico	2	18630	0.13	0	n/a	4	n/a	n/a	n/a
2196	Hz	4 Nov 2015	adult	Guanica, Puerto Rico	1	19857	0.06	0	n/a	n/a	1	0	n/a
2264	Hz	23 Oct 2015	adult	Juana Diaz, Puerto Rico	300	16526	21.3	1	3	n/a	n/a	n/a	n/a
2313	Hz	23 Mar 2015	adult	Juana Diaz, Puerto Rico	2	16338	0.14	0	n/a	5	n/a	n/a	n/a
2330	Hz	27 July 2015	adult	Juana Diaz, Puerto Rico	5520	19398	394	1	n/a	n/a	1	0	n/a
3513	Hz	1 July 2016	adult	Guanica, Puerto Rico	1	18882	0.06	0	n/a	n/a	1	0	n/a
3519	Hz	1 July 2016	adult	Guanica, Puerto Rico	2821	20322	176	1	n/a	n/a	1	0	n/a
3553	Hz	27 July 2016	adult	Juana Diaz, Puerto Rico	199	19119	12.3	1	n/a	n/a	1	0	n/a
4672	Hz	9 Feb 2016	adult	Manatee County, Florida, USA	1	17953	0.07	0	n/a	n/a	1	0	n/a
5111	Hz	26 July 2016	adult	Hamilton County, Florida, USA	548	12928	51	1	n/a	6	n/a	n/a	n/a
5112	Hz	26 July 2016	adult	Hamilton County, Florida, USA	553	17982	36.7	1	n/a	n/a	1	0	n/a
6793	Hz	27 Aug 2015	adult	Morrow County, Oregon, USA	77	18487	4.9	1	n/a	n/a	1	0	n/a
7049	Hz	29 Nov 2017	adult	Jackson County, Florida, USA	0	17773	0	0	n/a	n/a	1	0	n/a
7050	Hz	5 July 2016	adult	Jackson County, Florida, USA	1	17199	0.07	0	n/a	n/a	1	0	n/a
4643a	Hz	5 May 2016	adult	Manatee County, Florida, USA	2873	16968	218	1	1	n/a	n/a	n/a	n/a
4643b	Hz	5 May 2016	adult	Manatee County, Florida, USA	3503	16548	280	1	4	n/a	n/a	n/a	n/a
ard1	Hz	2015	adult	Larimer County, Colorado, USA	3	16898	0.21	0	5	n/a	n/a	n/a	n/a
a/z 1	Hz x Ha	2017	adult	USDA lab colony, Massachusetts, USA	0	19407	0.06	0	n/a	9	1	1	Acc#, #
a/z 14	Hz x Ha	2017	adult	USDA lab colony, Massachusetts, USA	1	17489	0.07	0	n/a	n/a	1	1	n/a
a/z 2	Hz x Ha	2017	adult	USDA lab colony, Massachusetts, USA	1	18839	0.06	0	n/a	10	1	1	n/a
a/z 3	Hz x Ha	2017	adult	USDA lab colony, Massachusetts, USA	2	18798	0.13	0	n/a	n/a	0	1	n/a
a/z 4	Hz x Ha	2017	adult	USDA lab colony, Massachusetts, USA	2	17587	0.13	0	n/a	n/a	0	1	n/a
a/z 5	Hz x Ha	2017	adult	USDA lab colony, Massachusetts, USA	0	17066	0	0	n/a	n/a	1	1	n/a

Supplementary Table S2 ddPCR results from bulk samples tested for prevalence of HzNV-2

Year collected	Life Stage	Origin	Positive droplets	Total droplets	Concentration copies/uL of PCR	HzNV-2 presence/ absence	Number of individuals in each bulk extraction
2019	adults	Texas	20	19679	1.2	1	3
2019	adults	Texas	1909	19528	121	1	14
2019	adults	Texas	2760	19908	176	1	3
2020	adults	Georgia	2	20870	0.11	0	10
2020	adults	Georgia	86	20697	4.9	1	40
2020	adults	Georgia	48	19542	2.9	1	27
2020	adults	Lousiana	2	17828	0.13	0	10
2020	adults	Lousiana	5911	20180	408	1	34
2020	adults	Lousiana	16	20542	0.92	0	3
2019	adults	Missouri	4	16840	0.28	0	40
2019	adults	Missouri	5	21025	0.28	0	47
2019	adults	Missouri	3099	21224	186	1	47
2020	adults	Indiana	2	20847	0.11	0	2
2020	adults	Indiana	13	19678	0.78	0	3
2020	adults	Indiana	1	18571	0.06	0	5
2020	adults	New Mexico	564	19989	33.7	1	33
2020	adults	New Mexico	1	17635	0.07	0	33
2020	adults	New Mexico	1	19050	0.06	0	33
2020	adults	Illinois	267	19555	16.2	1	33
2020	adults	Illinois	1192	19266	75.1	1	33
2020	adults	Illinois	0	8820	0	0	13
2019	adults	Mississippi	8260	20533	605	1	67
2019	adults	Mississippi	8	18664	0.5	0	67
2019	adults	Mississippi	2221	19979	139	1	67
2020	adults	Florida	748	8967	102	1	13
2020	adults	Florida	64	19282	3.9	1	27
2020	adults	Florida	1027	19953	62.2	1	7
2016	adults	Colorado	7683	19841	576	1	100
2016	adults	Colorado	5023	19162	358	1	200

Supplementary Table S3. Results of model tests for each gene cds alignment used in the DNA-based phylogenetic analysis.

ORF	AICc	BIC
PCFT	GTR+I+G	TVMef+I+G
TS	GTR+I+G	GTR+I+G
cSHMT	GTR+I+G	TrN+I+G
RNR	GTR+I+G	GTR+I+G

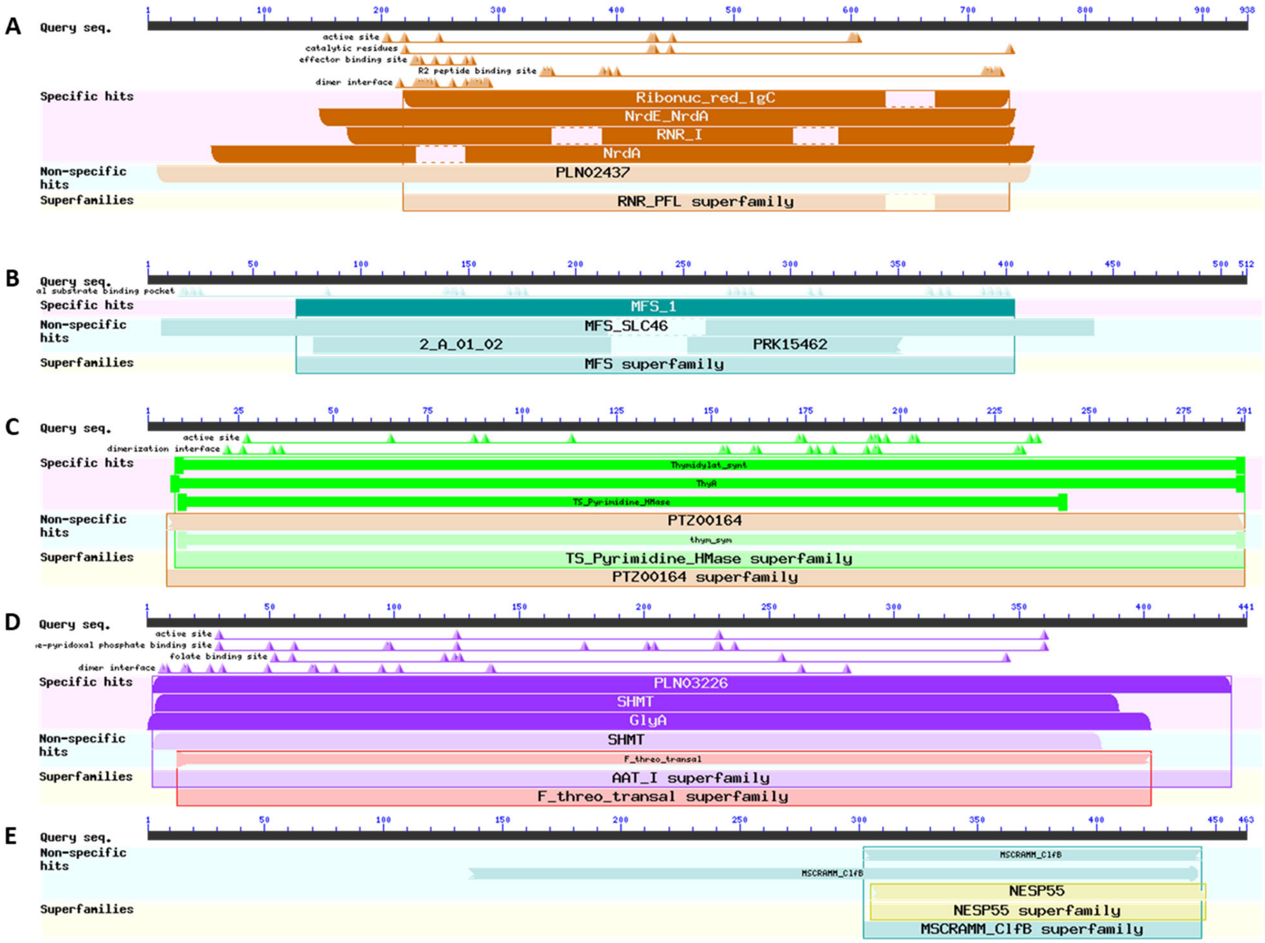

Figure S1. Translated HzNV-2 ORFs inferred to be recent host acquisitions BLASTP searched against the clustered nr database. A) ORF Hz2V047 annotated as Ribonucelotide reductase (RNR), B) ORF Hz2V066 annotated as Serine hydroxymethyltransferase (SHMT), C) ORF Hz2V023 annotated as Proton coupled folate transporter (PCFT), D) ORF Hz2V035 annotated as Thymidylate synthase (TS), and E) ORF Hz2V091annotated as bacterial cell surface protein.

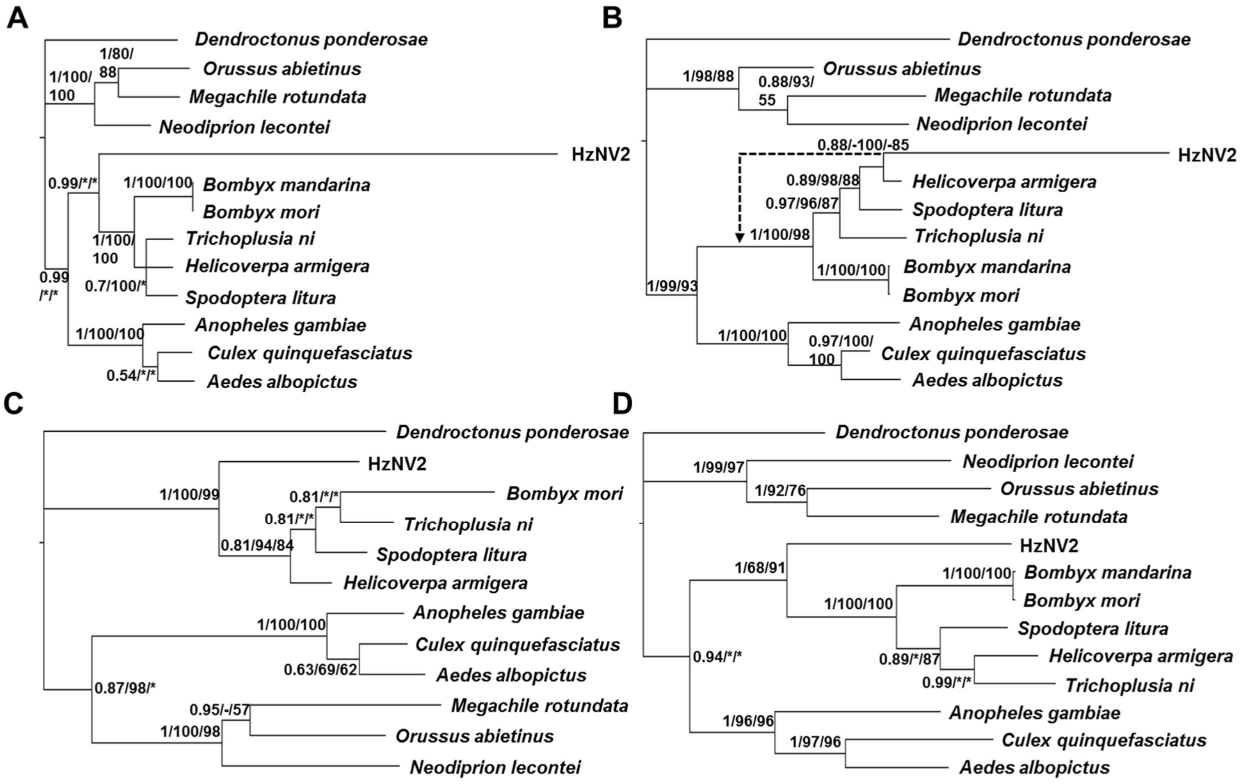

Figure S2. Phylogenetic analyses of sequences from A) RNR; B) cSHMT; C) PCFT; and D) TS. Numbers adjacent to nodes indicate posterior probability from Bayesian inference (BI), jackknife values from neighbor joining, and bootstrap values from the parsimony method. All trees shown in figure are from the BI results, * indicates collapse of branch in the corresponding method and – indicates different branching order. Different branching order and associated support values shown where relevant. *Dendroctonus ponderosae* was set as the outgroup in all analyses.

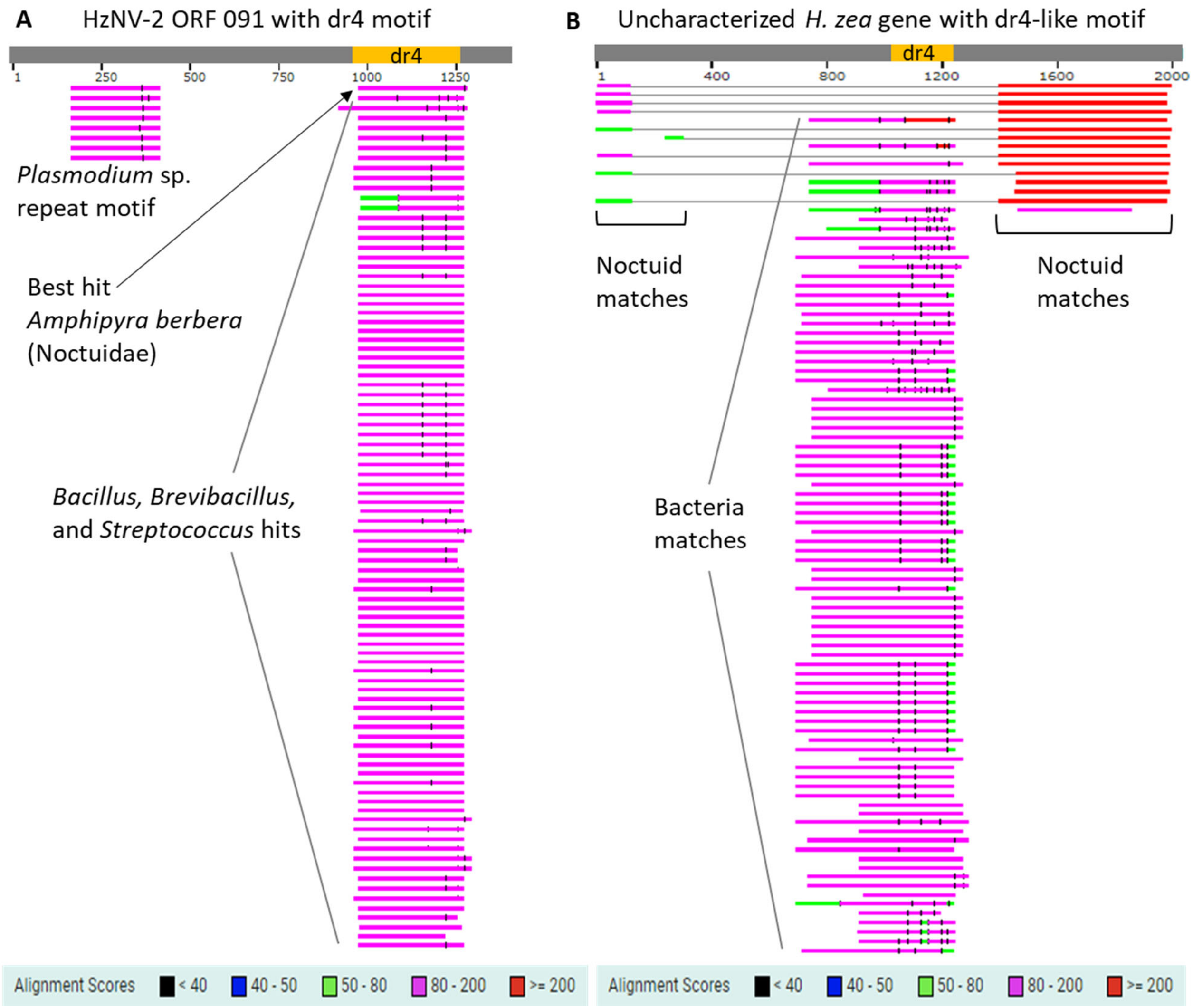

Figure S3. Best sequence matches mapped to A) HzNV-2 ORF Hz2V091 and B) an *H. zea* gene of unknown function with a central region similar to HzNV-2 dr4 in ORF Hz2V091.

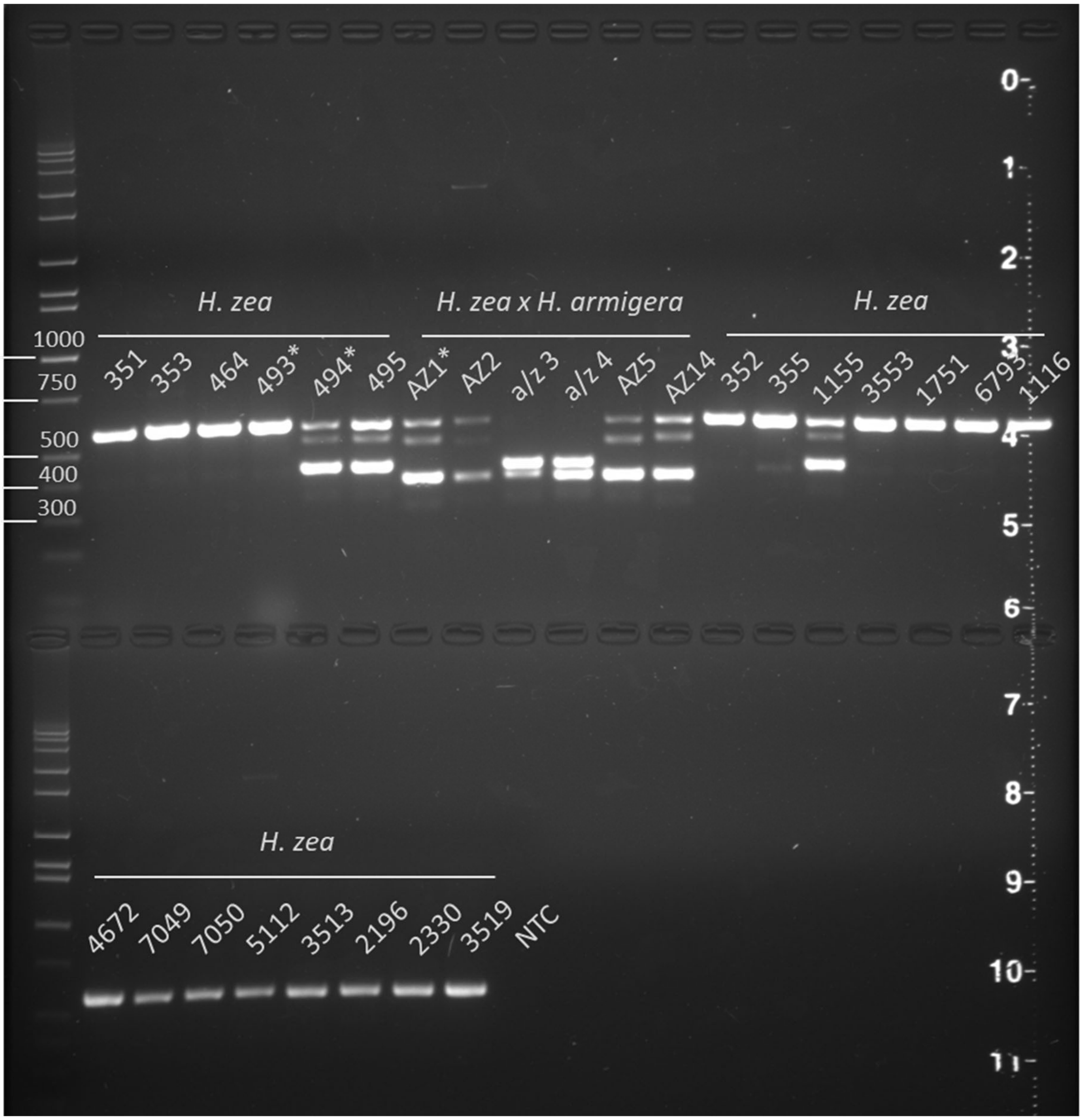

Figure S4. Agarose gel (1%) of PCR products spanning the TE insert in intron 7 of cSHMT from *H. zea*, and lab-reared hybrids. HzNV-2 test results on these samples can be found in Supplementary Table S1. * Refers to samples that were Sanger sequenced; accessions given in Table S1.

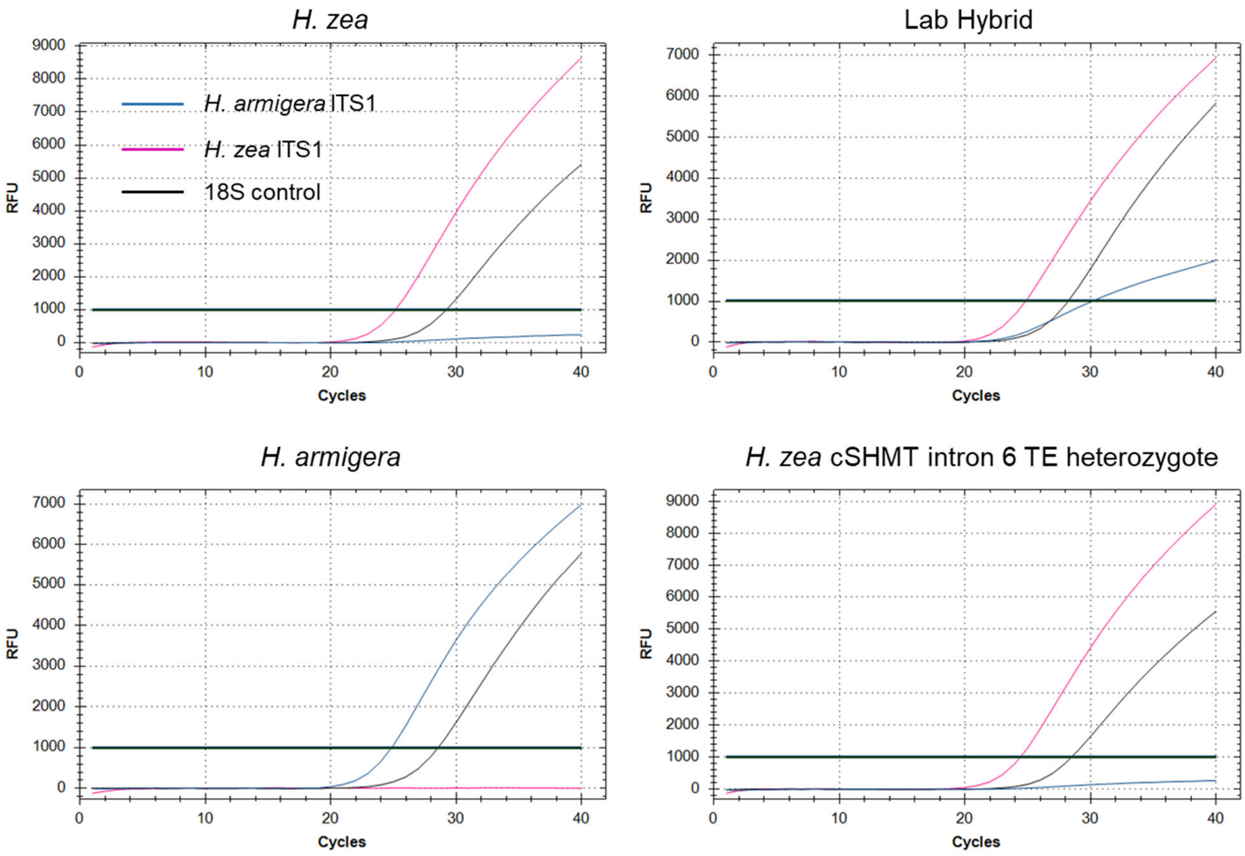

Figure S5. The cSHMT Intron 7 TE-inserted specimen with gel banding pattern similar to *H. zea* x *H. armigera* lab hybrid specimens but is identified as *H. zea* using the validated real-time PCR method from Gilligan et al. (2015).

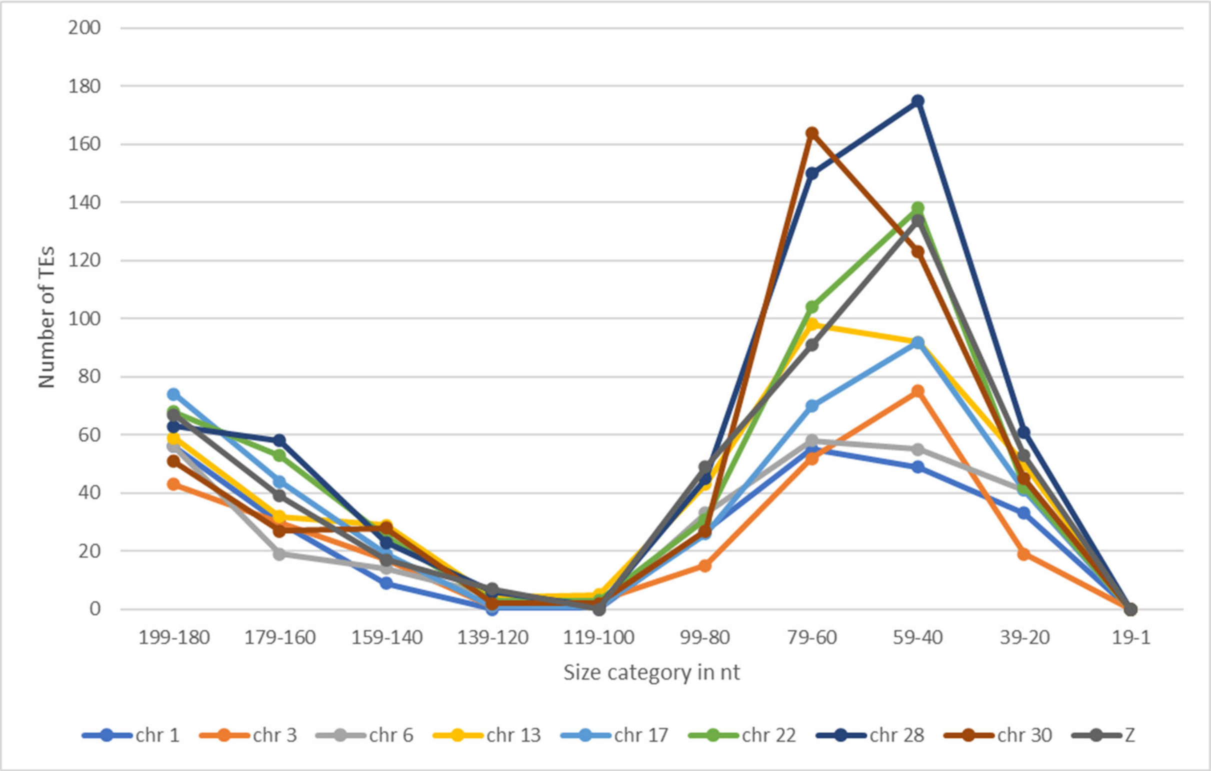

Figure S6. The size distribution of TEs with similar sequence identities (cutoff e-value ≤ 0.01) to those found in P450 genes and cSHMT intron 7 across a subset of chromosomes.

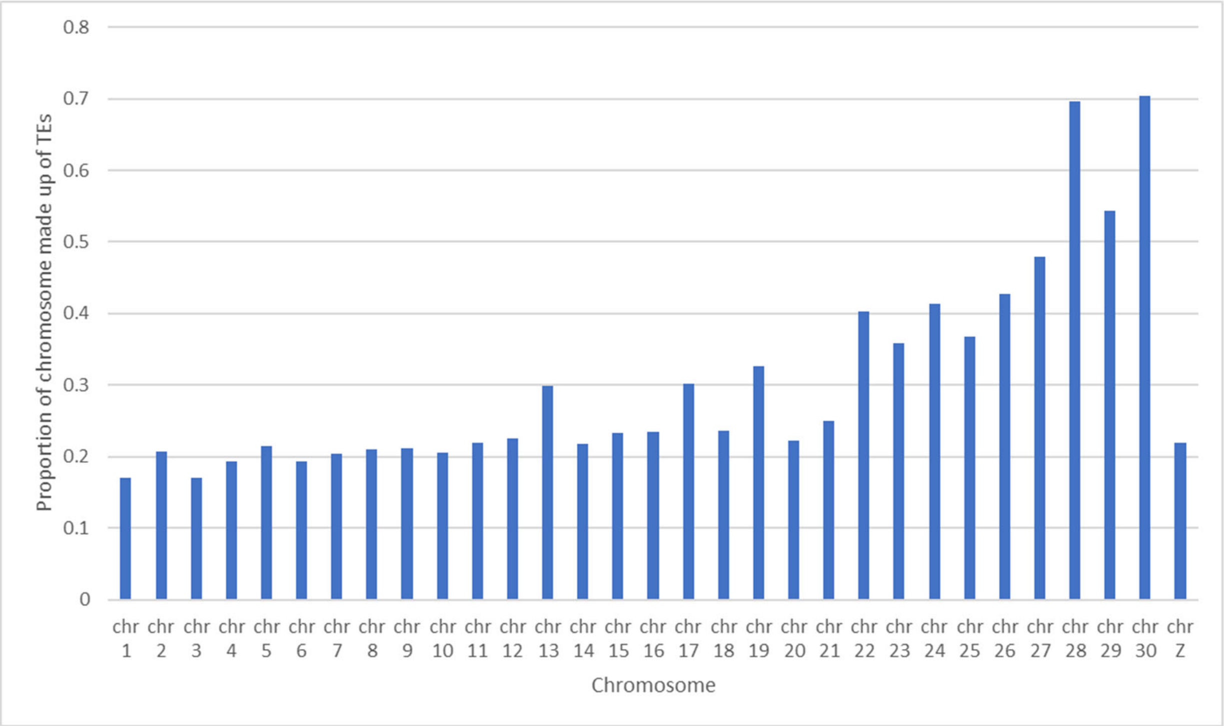

Figure S7. The proportion of each *H. zea* chromosome made up of TEs with similar sequence identities (cutoff e-value ≤ 0.01) to those found in P450 genes and cSHMT intron 7. Chromosomes ordered largest (15.51 Mb chr 1) to smallest (6.32 Mb chr 30) with the Z chromosome at 18.81 Mb listed last.

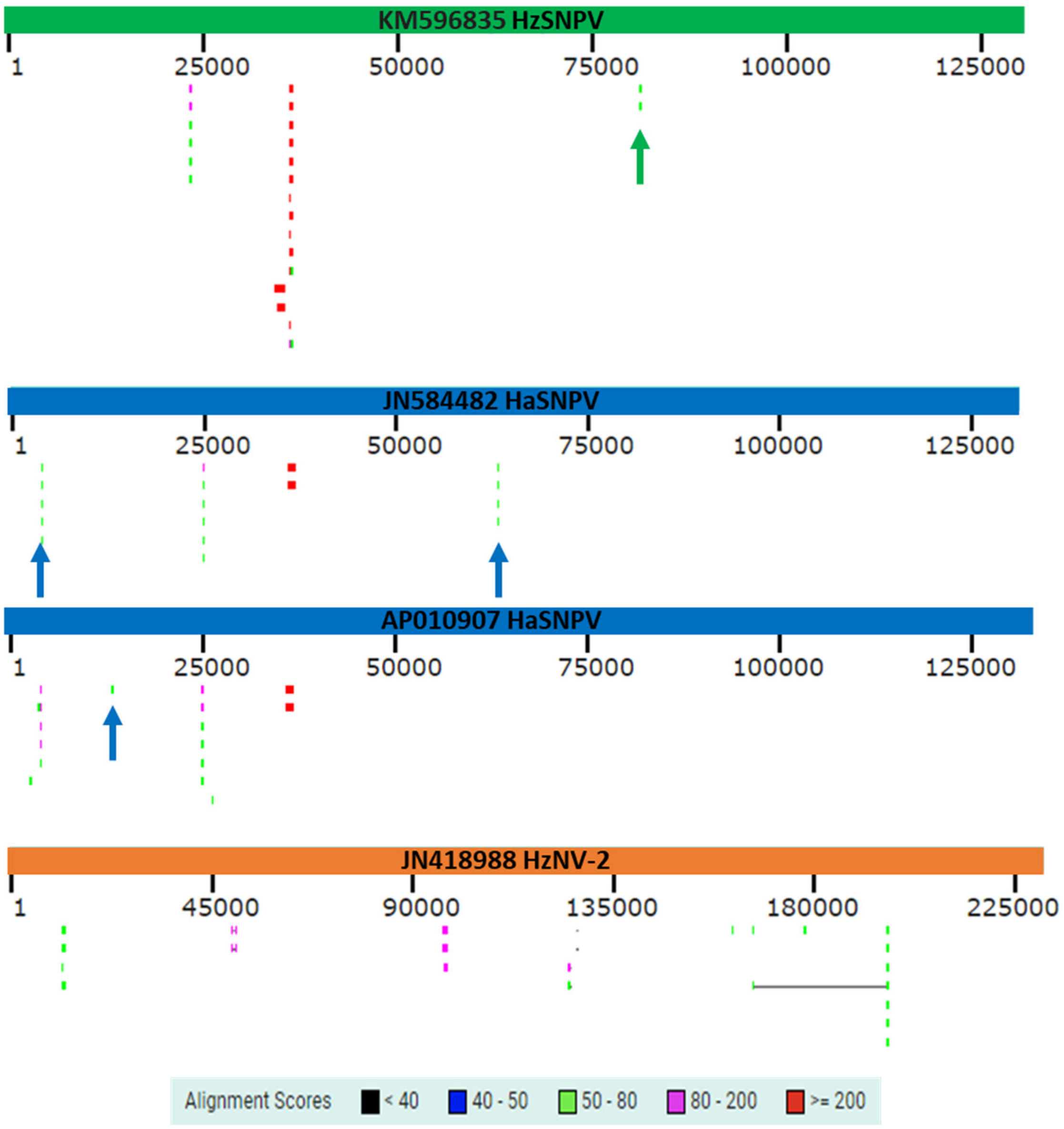

Figure S8. Inferred recent host DNA acquisitions mapped to baculoviruses HaSNPV (blue), HzSNPV (green), and nudivirus HzNV-2 (orange). Arrows indicate inferred host DNA acquisitions since *H. armigera* and *H. zea* divergence (for the baculoviruses only as the sister species of HzNV-2 has not been determined). All searches used complete viral genomes as queries to wgs *H. armigera* taxid 29058. Viral genomes not to scale.
